# Computational estimation of potential inhibitors from known drugs against the main protease of SARS-CoV-2†

**DOI:** 10.1039/d1ra02529e

**Published:** 2021-05-12

**Authors:** Nguyen Minh Tam, Minh Quan Pham, Nguyen Xuan Ha, Pham Cam Nam, Huong Thi Thu Phung

**Affiliations:** Computational Chemistry Research Group, Ton Duc Thang University Ho Chi Minh City Vietnam nguyenminhtam@tdtu.edu.vn; Faculty of Applied Sciences, Ton Duc Thang University Ho Chi Minh City Vietnam; Institute of Natural Products Chemistry, Vietnam Academy of Science and Technology Hanoi Vietnam; Graduate University of Science and Technology, Vietnam Academy of Science and Technology Hanoi Vietnam; Faculty of Chemistry and Environment, Thuyloi University, Ministry of Agriculture and Rural Development Hanoi Vietnam; Department of Chemical Engineering, The University of Da Nang, University of Science and Technology Da Nang City Vietnam; NTT Hi-Tech Institute, Nguyen Tat Thanh University Ho Chi Minh City Vietnam ptthuong@ntt.edu.vn

## Abstract

The coronavirus disease (COVID-19) pandemic caused by severe acute respiratory syndrome coronavirus 2 (SARS-CoV-2) has rapidly spread worldwide recently, leading to global social and economic disruption. Although the emergently approved vaccine programs against SARS-CoV-2 have been rolled out globally, the number of COVID-19 daily cases and deaths has remained significantly high. Here, we attempt to computationally screen for possible medications for COVID-19 *via* rapidly estimating the highly potential inhibitors from an FDA-approved drug database against the main protease (Mpro) of SARS-CoV-2. The approach combined molecular docking and fast pulling of ligand (FPL) simulations that were demonstrated to be accurate and suitable for quick prediction of SARS-CoV-2 Mpro inhibitors. The results suggested that twenty-seven compounds were capable of strongly associating with SARS-CoV-2 Mpro. Among them, the seven top leads are daclatasvir, teniposide, etoposide, levoleucovorin, naldemedine, cabozantinib, and irinotecan. The potential application of these drugs in COVID-19 therapy has thus been discussed.

## Abstract 2

Approved drugs predicted to interact with critical residues in the substrate-binding site of SARS-CoV-2 Mpro can be promising inhibitors.

## Graphical abstract

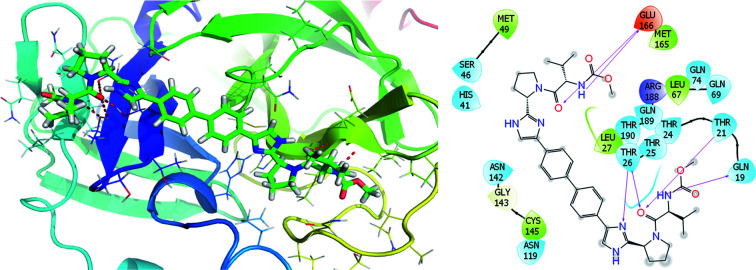

## Introduction

The ongoing deadly pneumonia disease (COVID-19) caused by severe acute respiratory syndrome coronavirus 2 (SARS-CoV-2) has unstoppably spread globally since its first identification in December 2019. In March 2020, the WHO (World Health Organization) classified the COVID-19 outbreak as a “Global Pandemic”.^1^ The transmission rate of the viral infection is extremely high^2^ while the fatality rate ranges from 1% to 12%.^3^ Recently, the COVID-19 disease has affected 221 countries and territories around the world with more than 153 million reported cases and 3.2 million deaths.^4^

SARS-CoV-2 has a single-stranded positive-sense RNA genome with a length of approximately 29.9 kb.^5,6^ The viral genome is composed of 11 open reading frames coding for more than 20 different proteins. Among them, the SARS-CoV-2 main protease (Mpro), one of the most important proteins during the viral translation, is required to digests polyproteins at eleven or more conserved cleavage sites to produce various functional proteins.^7^ The polypeptides generated are critical for the viral transcription and replication during its infection. The SARS-CoV-2 Mpro is well conserved in the Coronaviridae family^8^ while its closely related homologs in humans have lacked. These characteristics cause Mpro one of the most interesting targets for the selection of antiviral drugs to restrain the SARS-CoV-2 growth and replication.^9,10^ Consequently, multiple investigations have been done to define the promising inhibitors of this protease.^7,11–18^

Nowadays, the computer-aided drug design (CADD) approach has been broadly shown to remarkably save time and cost in the development of a new medication.^19,20^ In CADD, the ligand-binding free energy Δ*G* of an inhibitor with the targeted protein can be accurately predicted *via* molecular dynamics (MD) simulations.^19,20^ Accordingly, rapidly and precisely estimating the ligand-binding free energy is extremely essential for identifying potential inhibitors.^21,22^ Previously, the fast pulling of ligand (FPL) simulations was shown to effectively and accurately estimate the relative binding affinities of small molecules against HIV-1 (human immunodeficiency virus 1) protease or CHK1 (checkpoint kinase 1) with an affordable Central Processing Unit (CPU) time consumption.^23,24^ It is worthy to mention that the computational combination of molecular docking and the FPL approach was validated on available inhibitors of SARS-CoV-2 Mpro and showed good agreement between calculated binding free energies and experimental values.^13,14,25^

Computational drug repositioning has been a promising strategy to discover agents from known drugs for efficient response to the COVID-19 pandemic.^26–28^ Recently, 17 approved drugs have been identified as inhibitors of SARS-CoV-2 Mpro *via* molecular docking and molecular dynamics simulations.^29^ Artificial intelligence (AI) was also used to repurpose existing drugs.^30^ However, the binding free energy between ligands and SARS-CoV-2 Mpro was not calculated in those previous works, probably leading to insufficient binding affinities for drug repurposing of compounds identified. Notably, molecular docking and the FPL simulations were previously demonstrated that they could adopt high accurate results compared with the respective experiments.^14^ Therefore, in this study, the possible inhibitors of SARS-CoV-2 Mpro were screened from 2100 FDA-approved drugs using a combination of molecular docking and the FPL simulations. The results revealed that twenty-seven compounds could form large binding affinities to SARS-CoV-2 Mpro. These compounds thus become potential candidates for drugs against the COVID-19 disease effectively.

## Materials and methods

### Structure of SARS-CoV-2 Mpro and ligands

The crystal structure of SARS-COV-2 Mpro in monomeric form (6Y2F) was downloaded from the Protein Data Bank.^11^ The structure of ligands was obtained from the ZINC15 sub-database named FDA-approved drugs.^31^

### Molecular docking simulations

The Autodock Vina version 1.1 package was utilized to dock the screened ligands to the SARS-CoV-2 Mpro.^32^ The parameters of the molecular docking simulations were prepared by AutodockTools 1.5.6 (ref. 33) following the earlier studies.^34,35^ In detail, the exhaustiveness parameter of global searching that corresponds to the accuracy of the docking simulation was defined as 8 which represents default options. The atomic charges of protein and ligands were anticipated using the Gasteiger–Marsili approach.^36,37^ The protein and ligands were emblemed by a united atom model with explicit polar hydrogens.^38^ The maximum energy difference between the worst and best docking modes was set to 7 kcal mol^−1^. The grid center of Vina docking was selected as the center of mass of compound α-ketoamide 13b, which was obtained using the experimental pose.^11^ The highest binding affinity profiles were chosen as the best docking conformation. The docking grid was determined as 2.6 × 2.6 × 2.6 nm which is able to accommodate the whole targeted active site.^13–15^

### Molecular dynamics simulations

We used the GROMACS version 5.1.5 (ref. 39) to simulate the conformation change of the complex SARS-CoV-2 Mpro and ligand. The protein and ions were parameterized using the Amber99SBILDN force field.^40^ The water molecules were topologized *via* the TIP3P water model.^41^ The general Amber force field (GAFF)^42^ was applied to represent the ligand using AmberTools18.^43^ Combining the AMBER99SB-ILDN force field and water model provides one of the best options to estimate the free energy.^44,45^ The restrained electrostatic potential (RESP) method^46^ was used to fit the ligand atomic charges based on density functional theory (DFT) calculations using the combination of the B3LYP functional and 6-31G(d,p) basis set. The AMBER force field format was transformed to GROMACS using the ACPYPE protocol.^47^ The time steps of MD simulations were set to 2 fs. MD simulations were carried out with an integrator at the absolute temperature of 300 K controlled by V-rescale. The 0.9 nm cutoff was applied to the non-bonded atoms pair list. The steepest descent method was initially applied to the complex of SARS-CoV-2 Mpro and inhibitor to minimize the system. To relax the system, the 0.1 ns of NVT and 2.0 ns of NPT simulations were computed. During the NVT and NPT ensembles, the C_α_ atoms of SARS-CoV-2 Mpro were positionally restrained by a small harmonic force of 1000 kJ mol^−1^ nm^−2^. The solvated complexes were recorded every 10 ps over MD simulations.

### Fast pulling of ligand simulations

The last snapshot of NPT simulations was then applied as the starting conformation of the steered-MD (SMD) simulation.^23^ The complex of SARS-CoV-2 and ligand was covered into a rectangular periodic boundary conditions box (9.83 × 5.92 × 8.70 nm).^13,25^ More than 50 000 atoms including SARS-CoV-2 Mpro, ligand, water molecules and counter-balanced ions Na^+^ were involved in the simulated system. To pull the ligand out of the binding site in SARS-CoV-2 Mpro, an external harmonic force *F* = *k*(*νt* − *z*) along the *Z*-axis was applied on the center of mass of the ligand in the complex system (a spring constant of the cantilever of *k* = 0.005 nm ps^−1^, pulling speed of *ν* = 600 kJ mol^−1^ nm^−2^, and *z* is the displacement of the ligand mass center from its initial position).^23,25,48^ The work of external force *W* is calculated according to the following equation:1
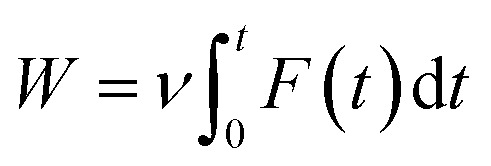


During SMD simulations, the solvated complex systems were recorded the external pulling force and the ligand displacement every 0.1 ps for the estimation of the ligand-binding affinity.^23^ For better sampling, 8 non-equilibrium MD simulation trajectories were independently conducted for each ligand–Mpro complex beginning with the same initial crystal structure but different random velocities. The mean external work 〈*W*〉 was averaged from 8 SMD trajectories for each complex.

### Analyzed tools

To predict the ligand protonation state, the Chemicalize tools (http://www.chemicalize.com), a website application of the ChemAxon, were utilized. A hydrogen bond (HB) is defined if the angle of an acceptor (A)–hydrogen (H)–donor (D) is larger than 135° with the distance from A to D is smaller than 0.35 nm. A sidechain contact is determined if the distance between non-hydrogen atoms of SARS-CoV-2 Mpro and the ligands is smaller than 0.45 nm. The interaction between the SARS-CoV-2 Mpro protein and the ligand was illustrated by the molecular modeling software Maestro (free version).^49^

## Results and discussion

### Molecular docking simulation

**Fig. 1:**
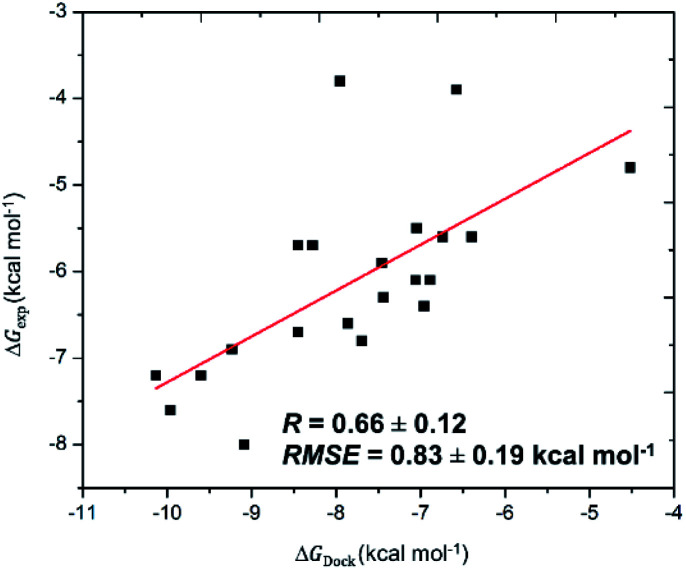
Correlation between molecular docking and experiment. The calculated errors are the standard error.

The SARS-CoV-2 Mpro in monomeric form can be used as a target for CADD to prevent the function of SARS-CoV-2 Mpro^13,14,25^ Autodock Vina,^32^ an open-source docking package, is one of the most popular docking protocols to rapidly estimate the binding affinity and binding pose of the protein and ligand complex. To validate the suitability of the approach, Autodock Vina was first applied to dock 21 SARS-CoV-2 Mpro inhibitors that have been confirmed experimentally. As was expected, the approach formed appropriate results for calculating the ligand-binding affinities of SARS-CoV-2 Mpro (Table 1) with a correlation coefficient of *R*_Dock_ = 0.66 ± 0.12 (Fig. 1), which is in good agreement with the recent work.^14^ Additionally, the root-mean-square error (RMSE) concerning practical data was calculated as RMSE = 0.83 ± 0.19 kcal mol^−1^. Noted that the results reported are consistent with the earlier studies.^14^

**Table 1:** The binding affinity values of verified SARS-CoV-2 Mpro inhibitors obtained *via* the docking simulations

No.	Inhibitor	Δ*G*_Dock_^a^	Δ*G*_exp_^b^
1	11a	−9.96	−7.60
2	11b	−10.13	−7.20
3	11r	−9.23	−6.90
4	13a	−7.70	−6.80
5	13b	−8.45	−6.70
6	Carmofur	−7.86	−6.60
7	Disulfiram	−6.89	−6.10
8	Ebselen	−8.45	−5.70
9	PX-12	−6.39	−5.60
10	Shikonin	−6.58	−3.90
11	Tideglusib	−7.95	−3.80
12	Digitoxin	−9.09	−8.00
13	Oubain	−9.6	−7.20
14	Remdesivir	−6.96	−6.40
15	Oxyclozanide	−7.44	−6.30
16	Ebastine	−7.06	−6.10
17	Toremifene	−7.46	−5.90
18	Hexachlorophene	−8.28	−5.70
19	Chloroquine	−6.74	−5.60
20	Triparanol	−7.05	−5.50
21	Favipiravir	−4.52	−4.80

a The docking scores Δ*G*_Dock_ were obtained *via* the Autodock Vina package.

b The experimental binding free energy Δ*G*_exp_ was roughly identified using the reported values^50^ of inhibition constant IC_50_ with an assumption that IC_50_ is equal to *k*_i_. The unit is in kcal mol^−1^.

Based on the above results, the binding affinities between the SARS-CoV-2 Mpro and the screened ligands were first predicted *via* Autodock Vina. The docking results revealed that the calculated binding free energies for all computed complexes varied from −2.4 to −9.2 kcal mol^−1^. The average of binding free energies obtained of −5.99 kcal mol^−1^ with the standard error of the mean of 1.08 kcal mol^−1^. Based on the results, 27 top-lead ligands having the binding energy with SARS-CoV-2 Mpro more negative than −8.1 kcal mol^−1^ (Table 2) were chosen to be further examined by the FPL simulations.

**Table 2:** Twenty-seven top-lead compounds obtained by docking and FPL simulations

No.	Name	ZINC ID	Binding energy (kcal mol^−1^)	Average force (pN)	Average work (kcal mol^−1^)	Predicted Δ*G*^FPL^_Pre_
1	**Indocyanine green acid form**	**ZINC000008101127**	**−8.6**	**861.75 ± 42.65**	**94.55 ± 5.76**	**−10.81**
2	**Daclatasvir**	**ZINC000068204830**	**−8.1**	**590.59 ± 39.59**	**85.57 ± 6.52**	**−10.30**
3	**Teniposide**	**ZINC000004099009**	**−8.4**	**636.29 ± 28.39**	**71.74 ± 3.17**	**−9.53**
4	**Etoposide**	**ZINC000003938684**	**−8.1**	**652.51 ± 37.03**	**67.02 ± 4.55**	**−9.27**
5	**Levoleucovorin**	**ZINC000009212427**	**−8.1**	**610.87 ± 12.16**	**66.36 ± 1.89**	**−9.23**
6	**Naldemedine**	**ZINC000100378061**	**−8.2**	**648.26 ± 36.79**	**66.14 ± 3.12**	**−9.22**
7	**Cabozantinib**	**ZINC000070466416**	**−8.1**	**621.70 ± 28.96**	**62.88 ± 2.86**	**−9.03**
8	**Irinotecan**	**ZINC000001612996**	**−9.0**	**625.12 ± 41.31**	**62.15 ± 4.92**	**−8.99**
9	Azilsartan medoxomil	ZINC000014210642	−8.2	550.68 ± 22.92	58.67 ± 3.46	−8.80
10	Ergotamine	ZINC000052955754	−8.4	576.51 ± 25.68	58.63 ± 2.61	−8.80
11	Cromolyn	ZINC000253632968	−8.1	551.18 ± 37.07	55.90 ± 5.61	−8.64
12	Glecaprevir	ZINC000164528615	−8.5	551.22 ± 40.48	55.54 ± 3.99	−8.62
13	Dolutegravir	ZINC000058581064	−8.2	559.65 ± 24.05	55.05 ± 2.07	−8.59
14	Saquinavir	ZINC000029416466	−8.1	514.50 ± 31.70	54.97 ± 5.12	−8.59
15	Dihydroergotamine	ZINC000003978005	−8.7	543.99 ± 42.88	54.42 ± 5.10	−8.56
16	Accolate	ZINC000000896717	−8.7	498.69 ± 32.54	52.91 ± 4.84	−8.47
17	Lumacaftor	ZINC000064033452	−8.5	528.13 ± 36.91	50.37 ± 3.87	−8.33
18	Lifitegrast	ZINC000084668739	−8.3	521.70 ± 22.03	49.47 ± 2.43	−8.28
19	Doxazosin	ZINC000094566092	−8.1	524.16 ± 17.71	48.82 ± 2.18	−8.25
20	Rifaximin	ZINC000169621200	−8.3	530.94 ± 42.43	45.23 ± 5.55	−8.04
21	Ceftaroline	ZINC000003989268	−8.2	420.93 ± 53.33	44.59 ± 5.96	−8.01
22	Dutasteride	ZINC000003932831	−8.1	499.62 ± 22.11	43.85 ± 2.42	−7.97
23	Imatinib	ZINC000019632618	−8.3	474.81 ± 16.15	43.26 ± 3.02	−7.93
24	Raltegravir	ZINC000013831130	−8.1	487.90 ± 22.66	43.05 ± 1.93	−7.92
25	Trypan blue	ZINC000169289767	−9.2	409.97 ± 45.04	41.79 ± 7.06	−7.85
26	Nilotinib	ZINC000006716957	−8.5	486.41 ± 26.65	39.98 ± 2.79	−7.75
27	Regorafenib	ZINC000006745272	−8.3	425.92 ± 16.90	37.54 ± 2.99	−7.61

### Docking binding pose

**Fig. 2:**
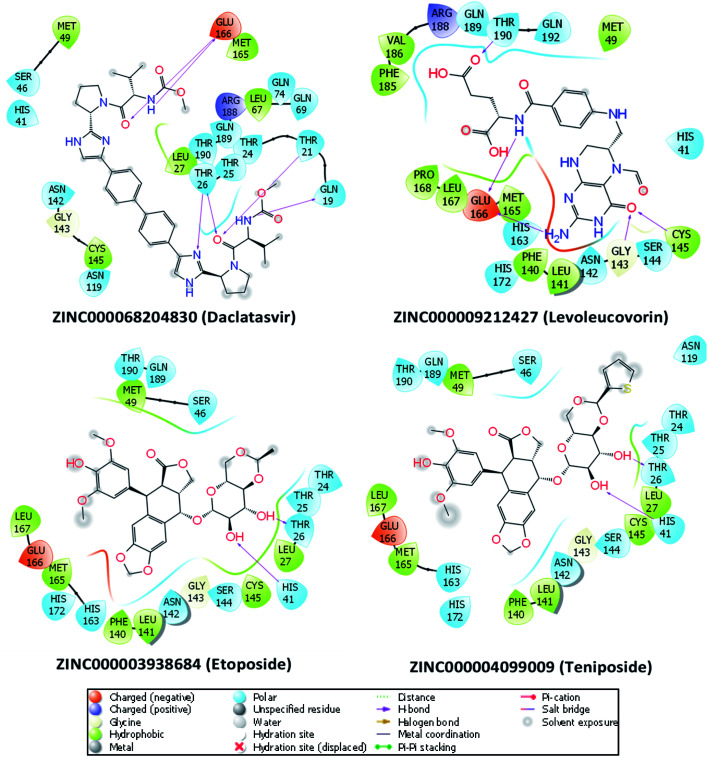
The detailed interactions between SARS-CoV-2 Mpro and four drugs obtained *via* molecular docking simulations are illustrated by the molecular modeling software Maestro (free version).^49^ HBs formed by residues of SARS-CoV-2 Mpro and ligands are indicated by purple arrows. Atoms of carbon, oxygen, nitrogen, and sulfur are presented in black, red, blue, and yellow, respectively.

To investigate the binding interaction between the 27 top-lead compounds and SARS-CoV-2 Mpro, a detailed analysis of their possible docked conformation was performed to explore their interaction with the SARS-CoV-2 Mpro-binding pocket. Accordingly, the preferential binding pose of top-lead drugs obtained by docking simulations in the complex with SARS-CoV-2 Mpro was determined. The detailed interaction of SARS-CoV-2 Mpro and the full top-lead compounds are illustrated in Fig. S1.† In particular, Fig. 2 shows the particular binding of representative drugs including daclatasvir, teniposide, etoposide, and levoleucovorin with SARS-CoV-2 Mpro. Note that the substrate-binding site of SARS-CoV-2 Mpro resides in a cleft between domain I and domain II.^11,51^ The obtained results suggest that the top-lead drugs identified by the docking method bind to the substrate-binding cleft of SARS-CoV-2 Mpro *via* different HBs. These compounds establish the sidechain interactions with a set of critical residues, including Thr26, His41, Leu141, Gly143, Ser144, Cys145, His163, Glu166, and Gln189, of SARS-CoV-2 Mpro. It should be noted that His41 and Cys145 reside in the Cys–His catalytic dyad and Glu166 is essential for dimerization of SARS-CoV-2 Mpro.^7,11^

### FPL simulation

**Fig. 3:**
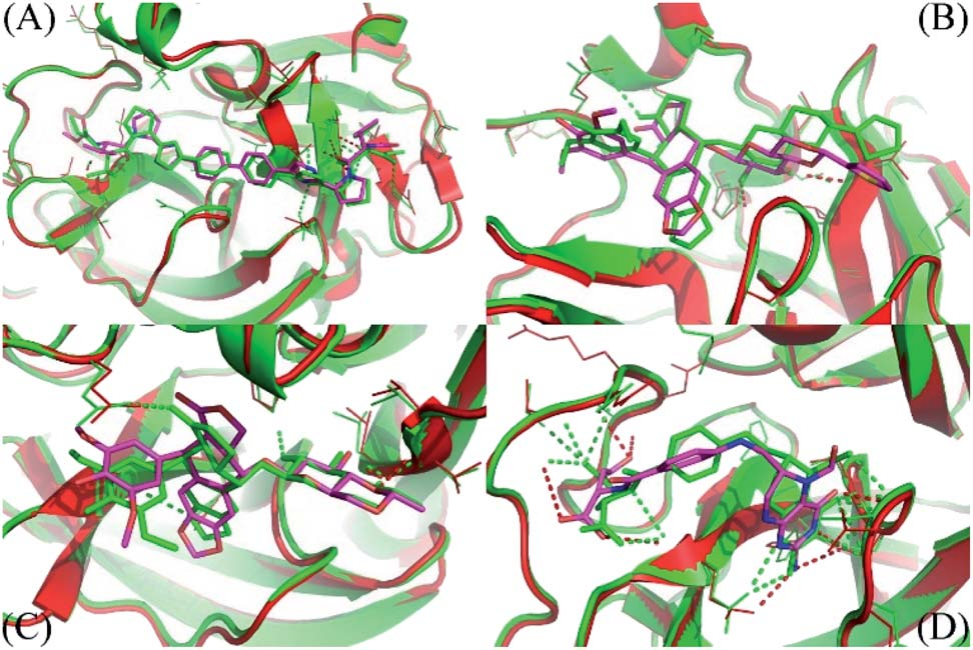
The comparison between MD refined conformations of the complexes and docked structures. The MD refined structure was obtained by all-atom clustering with a cut-off of 0.3 nm over the last NPT snapshots. (A) Is the daclatasvir complex; (B) is the teniposide system; (C) is the etoposide complex; (D) is the levoleucovorin system. Both receptors and ligands obtained from docking are displayed in green. The figure was generated using PyMOL 1.3 open source.

The molecular docking simulations were performed with numerous limitations such as inflexible receptor and small number trial positions of inhibitors, the MD/SMD simulations were thus executed to refine the outcomes.^52,53^ In this context, the FPL simulations were manipulated to re-rank the ligand-binding affinity to the SARS-CoV-2 Mpro since the approach successfully formed an appropriate outcome compared with the respective experiments, *R* = 0.76.^14^ During MD/SMD calculations, the complexes were first relaxed to the equilibrated states. The clustering method was then employed to estimate the stabilized structures of the complexes of SARS-CoV-2 Mpro and ligands with an all-atom root-mean-square deviation (RMSD) cutoff of 3.0 Å. The dominant conformations of the top-lead compounds in the complex with SARS-CoV-2 Mpro are depicted in Fig. S2.† Since changing from the implicit solvent environment (docking results) to the explicit solvent modeling (MD simulations), the complex structures were slightly refined with an averaged RMSD between the initial and MD refine of ligands of *ca.* 2.0 Å. The structural changes of representative compounds are described in Fig. 3. Although the value of RMSD is small, implying the success of the docking calculation, some charged groups of the ligands were also rotated and then formed HBs to the receptor. The shifts are small but important, resulting in the difference of the affinity ranking order of ligands (*cf.*Table 2).

Over the FPL simulations, the maximal value of external force called rupture force and the pulling work averaged from 8 independent SMD simulation trajectories were both used as criteria to rank the ligand affinity. However, the pulling work is more appropriate than the rupture force because it directly associates with the ligand-binding free energy *via* isobaric–isothermal Jarzynski's equality.^23^ The average of pulling forces of all complexes between SARS-CoV-2 and tested ligands in a time-dependent manner is shown in Fig. S3.† The obtained results indicated that the mean rupture forces ranged from 409.97 ± 45.04 (trypan blue free acid) to 861.75 ± 42.65 (indocyanine green acid form) pN with the average value of 552 ± 31.4 pN (Table 2). The pulling work was shown to be a key factor representing the binding of ligands and the protein.^23,24^ According to FPL calculations, the average pulling works of the tested 27 compounds fell within a range of 37.54 ± 2.99 (regorafenib) to 94.55 ± 5.76 (indocyanine green acid form) kcal mol^−1^ with the mean value of 55.94 ± 3.02 kcal mol^−1^ (Table 2).

Every complex of SARS-CoV-2 Mpro and ligand was computed over 8 independent FPL simulations which initiated from the same conformation but different in random velocity. One FPL trajectory consists of 0.1 ns of NVT, 2.0 ns of NPT, and 0.5 ns of SMD simulations. Totally, 20.8 ns of MD simulations were computed to evaluate the ligand-binding affinity with SARS-CoV-2 Mpro. The binding affinity of a ligand to the SARS-CoV-2 Mpro can thus be simulated 8 times during approximately 6 hours. Consequently, without the requirement of a professional computing system, the low CPU consumption allows the precise evaluation of the binding affinity of various compounds with SARS-CoV-2 Mpro at an appropriate time.

Recently, the FPL calculations have been proved to adopt a good agreement with the experimental data for SARS-CoV-2 Mpro and its inhibitors.^13,14^ The estimated binding free energies Δ*G*^FPL^_Pre_ between top-lead ligands and SARS-CoV-2 Mpro were thus calculated in the same way as in the previous study.^14^ The obtained results are shown in Table 2. The ligand with the predicted Δ*G*^FPL^_Pre_ more negative than −9.0 kcal mol^−1^ is thus strongly expected to be capable of inhibiting the function of the SARS-CoV-2 Mpro protein. Noted that indocyanine green acid form is a fluorescent dye used in medical diagnostics as an indicator substance in cardiac, circulatory, hepatic, and ophthalmic conditions^54^ which might not be suitable to serve as a drug for COVID-19 treatment. Consequently, seven approved drugs including daclatasvir, teniposide, etoposide, levoleucovorin, naldemedine, cabozantinib, and irinotecan were predicted as the very promising inhibitors of SARS-CoV-2 Mpro in practice due to their high binding affinities calculated (Table 2).

### The most potential inhibitors of SARS-CoV-2 Mpro identified

**Fig. 4:**
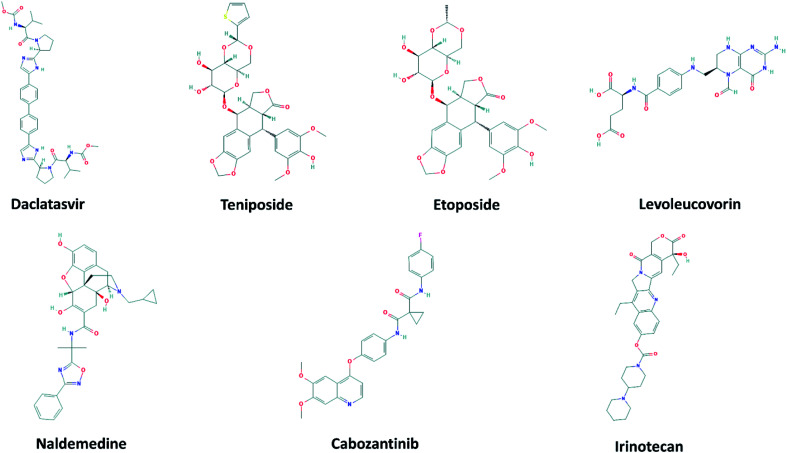
2D structure of potential inhibitors for SARS-CoV-2 Mpro predicted by molecular docking and FPL simulations from the ZINC15 sub-database named FDA-approved drugs. The 2D structures were downloaded from an open chemistry database PubChem.^60–66^

The 2D structures of 7 top-lead ligands of SARS-CoV-2 Mpro ranked by the FPL simulations are illustrated in Fig. 4. Except for levoleucovorin and cabozantinib of which molecular structures are considered as flexible, the other 5 compounds possess the rigid structure and thus, lacking molecular flexibility. However, the substrate-binding cleft on the surface of SARS-CoV-2 Mpro is expected to be flexible to sizably accommodate a broad type of compound. Importantly, all of the 7 top-lead inhibitors predicted have many sites to form intermolecular HBs with the protein, hence being able to strongly interact with SARS-CoV-2 Mpro.

Daclatasvir belonging to a valine and derivatives group is a direct-acting antiviral agent against Hepatitis C Virus (HCV), a positive-sense single-stranded RNA virus.^55^ Previously, the anti-HCV drug was shown to be effective in the treatment of Middle East Respiratory Syndrome (MERS) coronavirus.^56^ Therefore, anti-HCV drugs are expected to express a common antiviral activity against human coronaviruses. From our calculation, daclatasvir showed the highest affinity to SARS-CoV-2 Mpro among tested compounds (*cf.*Table 2) *via* forming HBs with a critical residue Glu166 and other residues, namely Thr21 and Thr26 (Fig. 2). Notably, it was shown that the combined use of sofosbuvir and daclatasvir improved the survival and clinical recovery of COVID-19 patients with modest to intense symptoms.^57^ The introduction of sofosbuvir and daclatasvir to standard care could reduce the hospitalization time for COVID-19 patients in comparison to standard care alone.^58,59^ These results indicated that daclatasvir is very promising in the treatment of COVID-19 patients and that our approach successfully selected the practically potential drug for COVID-19 therapy.

It is believed that both virus-infected and cancer cells require the elevation of nucleic acids and protein synthesis and energy metabolism. Thus, drugs inhibiting cancer cells can be effective in the suppression of viral replication. Indeed, different antineoplastic agents re-purposed for COVID-19 therapy have been applied for early clinical trials.^67,68^ Etoposide and teniposide are anticancer drugs for the treatment of several types of tumors, leukemia, and lymphoma.^69^ Etoposide and teniposide are both semisynthetic analogs of podophyllotoxin. They share a similar basic structure of the parent podophyllotoxin while the carbohydrate moiety of a methyl group in etoposide is substituted for a thenylidene group in teniposide,^69^ inducing a slight difference in binding affinities to SARS-CoV-2 Mpro (*cf.*Table 2). These two compounds are estimated to establish HBs with key residues Cys145 and His41 and residue Thr26 (Fig. 2). Teniposide possesses a log *P* value of 2.78 (DrugBank, Accession Number DB00444) while etoposide has a lower log *P* value of 1.16 (DrugBank, Accession Number DB00773). These values are in the optimal range of 1 to 3 for a compound to achieve appropriate physicochemical characteristics.^70^ However, teniposide which is predicted to have a higher affinity to SARS-CoV-2 Mpro has better membrane permeability. Irinotecan is indicated for colorectal and pancreatic cancer treatment or combined with cisplatin for the cure of small cell lung cancer.^71–73^ The Sn38 moiety is believed to mainly impact the high binding affinity of irinotecan to SARS-CoV-2 Mpro. The compound is predicted to form HBs with residues Thr24 and Thr26 of SARS-CoV-2 Mpro (Fig. S1†). Irinotecan (DrugBank, Accession Number DB00762) has a similar log *P* value and molecular weight to teniposide, indicating a comparable membrane permeability. In general, these three anti-cancer drugs are highly promising in the treatment of COVID-19.

Levoleucovorin, a folate analog, is utilized in rescue therapy to recover cells from the toxic effects of folate antagonists such as methotrexate after high-dose treatment in osteosarcoma therapy.^74^ Naldemedine is an opioid receptor antagonist and used for the treatment of opioid-induced constipation.^75^ Levoleucovorin and naldemedine share similar estimated binding affinities to SARS-CoV-2 Mpro (*cf.*Table 2). While naldemedine is predicted to form HBs with residues Thr24 and Ser46 (Fig. S1†), levoleucovorin is expected to establish several HBs with both critical residues Cys145 and Glu166 and other residues including Leu141, Gly143, Ser144, and Thr190 (Fig. 2). Nevertheless, levoleucovorin is hydrophilic with a low log *P* value of −2.8 (DrugBank, Accession Number DB11596), pointing out that the compound is not suitable as a drug for COVID-19 treatment. Meanwhile, naldemedine shows a log *P* value of 2.43, indicating its good ability to be transported through a cellular membrane.

Cabozantinib suppresses metastasis and oncogenesis by inhibiting receptor tyrosine kinases.^76^ Recently, several kinase inhibitors have been re-purposed for COVID-19 therapy.^77,78^ The molecular structure of cabozantinib is not too bulky, flexible, and contains fluoro, an element that can be found in many bioactive compounds. Cabozantinib is predicted to establish HBs with residues Ser46, Gly143 and a key residue Glu166 of SARS-CoV-2 Mpro (Fig. S1†). The high log *P* value 4.66 of cabozantinib (DrugBank, Accession Number DB08875) indicating the considerable level of toxicity may limit the application of this drug in practical use.

## Conclusion

Since the COVID-19 pandemic spread throughout the world, several inhibitors of SARS-CoV-2 Mpro have been identified experimentally.^11,79^ Based on CADD, various computational studies have also been conducted to search for the promising inhibitors of Mpro and other critical enzymes of SARS-CoV-2.^13,14,80–84^ Previously, a combination of molecular docking and FPL simulations was proved to efficiently predict the binding affinity of a ligand to SARS-CoV-2 Mpro. In this study, the same approach was employed to estimate the promising inhibitors for SARS-CoV-2 Mpro from a set of 2100 FDA-approved drugs. The binding conformation of the top-lead compounds identified with SARS-CoV-2 Mpro was also analyzed. The detailed interactions of the seven top-lead drugs including daclatasvir, teniposide, etoposide, levoleucovorin, naldemedine, cabozantinib, and irinotecan that have the predicted binding free energies with SARS-CoV-2 Mpro less than −9.00 kcal mol^−1^ indicated that these drugs all occupied the substrate-binding pocket of SARS-CoV-2 Mpro and thus potentially hindered the protease activity of the enzyme. These drugs interact with important residues of SARS-CoV-2 Mpro including Thr26, His41, Leu141, Gly143, Ser144, Cys145, His163, Glu166, and Gln189. Further *in vitro* and *in vivo* investigations are needed to be performed to validate the obtained results.

## Conflicts of interest

The authors declared that they have no conflicts of interest to this work.

## Supplementary Material

RA-011-D1RA02529E-s001
