# Supplementary material for: Computational estimation of potential inhibitors from known drugs against the main protease of SARS-CoV-2
Source: RSC Adv. 2021 May 12;11(28):17478–86. doi: 10.1039/d1ra02529e (PMC9032918; doi:10.1039/d1ra02529e)
Supplement: RA-011-D1RA02529E-s001 [file RA-011-D1RA02529E-s001.pdf]

Electronic Supplementary Material (ESI) for RSC Advances.
This journal is © The Royal Society of Chemistry 2021

Supplementary Data

Computational Estimation of Potential Inhibitors from the Known Drugs against the Main Protease of SARS-CoV-2

Nguyen Minh Tam[a,b*], Minh Quan Pham[c,d], Nguyen Xuan Ha[e], Pham Cam Nam,[f] Huong Thi Thu Phung[g*]

[a]*Computational Chemistry Research Group, Ton Duc Thang University, Ho Chi Minh City, Vietnam. Email: nguyenminhtam@tdtu.edu.vn*

[b]*Faculty of Applied Sciences, Ton Duc Thang University, Ho Chi Minh City, Vietnam.*

[c]*Institute of Natural Products Chemistry, Vietnam Academy of Science and Technology, Hanoi, Vietnam*

[d]*Graduate University of Science and Technology, Vietnam Academy of Science and Technology, Hanoi, Vietnam*

[e]*Faculty of Chemistry and Environment, Thuyloi University, Ministry of Agriculture and Rural Development, Hanoi, Vietnam*

[f]*Department of Chemical Engineering, The University of Da Nang-University of Science and Technology, Da Nang City, Vietnam*

[g]*NTT Hi-Tech Institute, Nguyen Tat Thanh University, Ho Chi Minh City, Vietnam. Email: ptthuong@ntt.edu.vn*

ZINC000169289767	ZINC000001612996

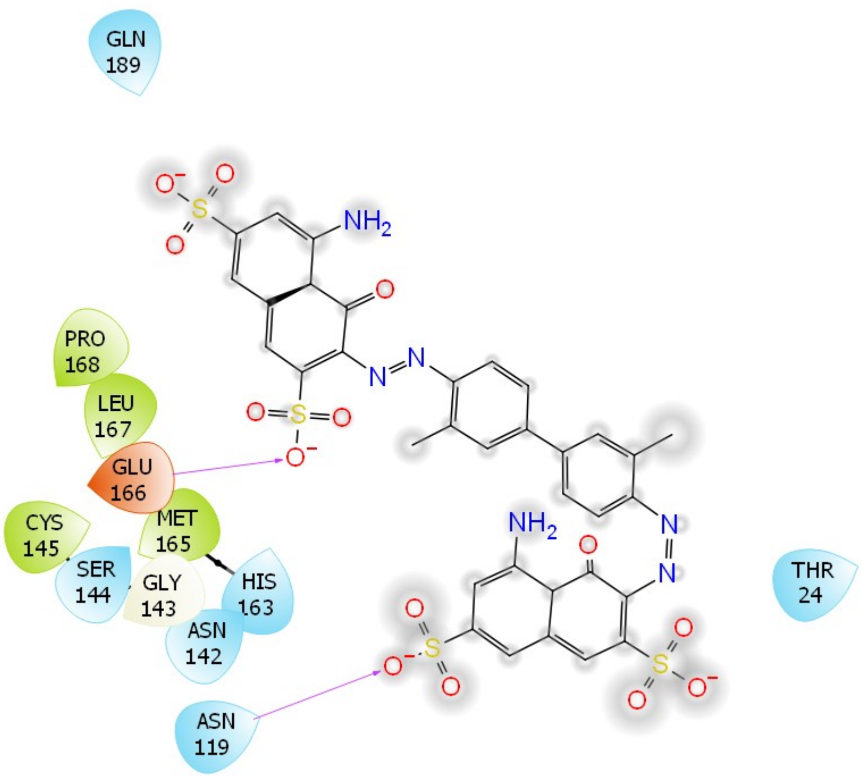

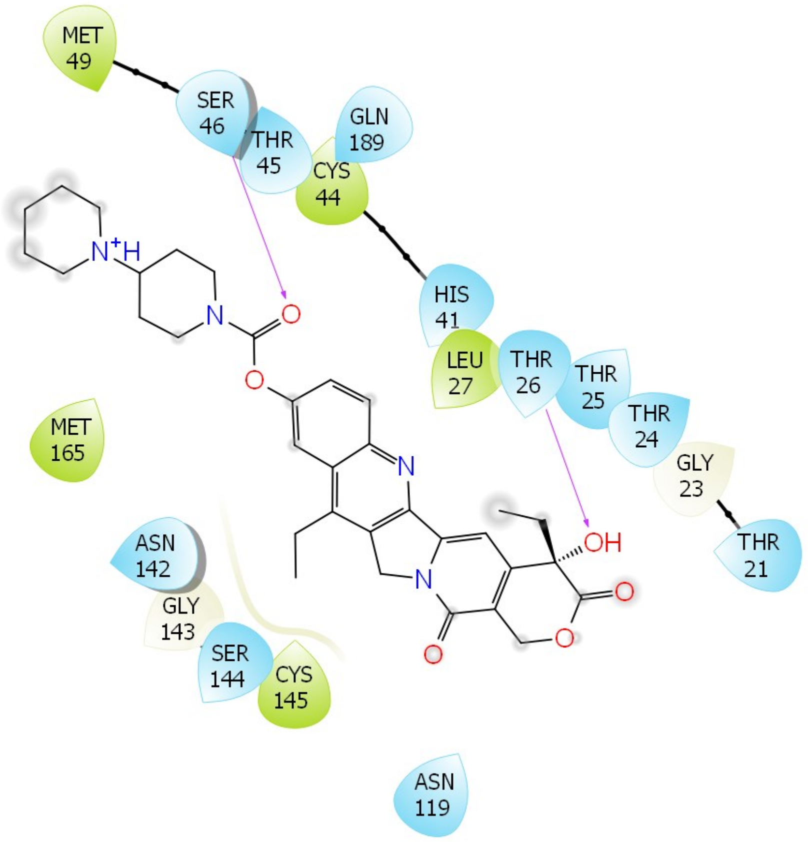

ZINC000000896717

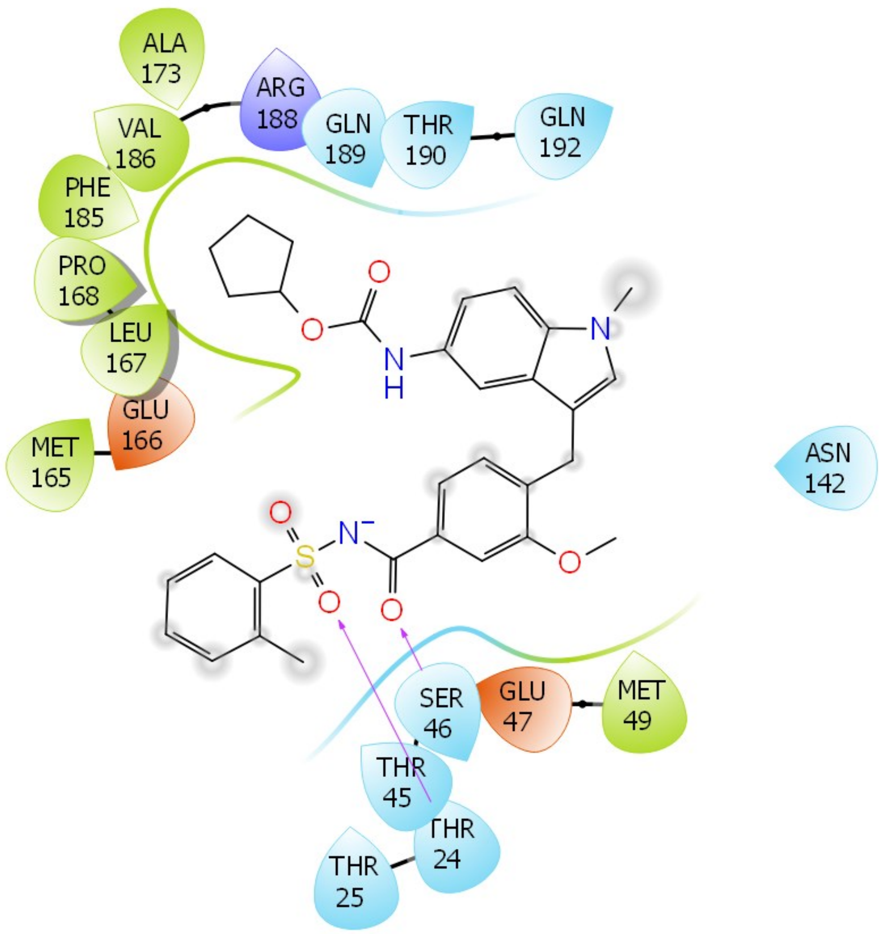

ZINC000003978005

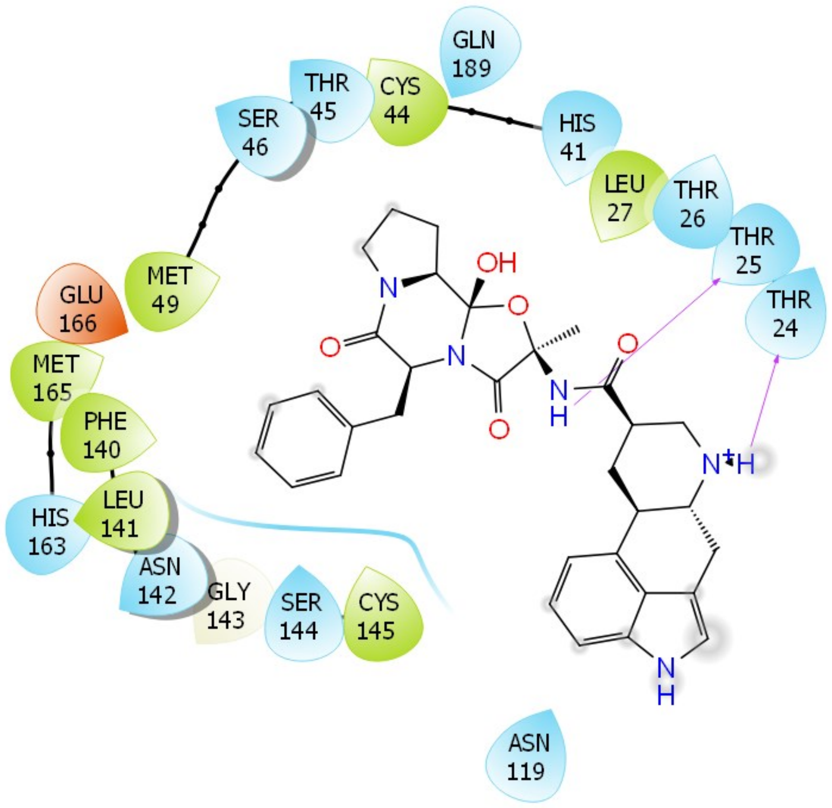

ZINC000008101127	ZINC000006716957
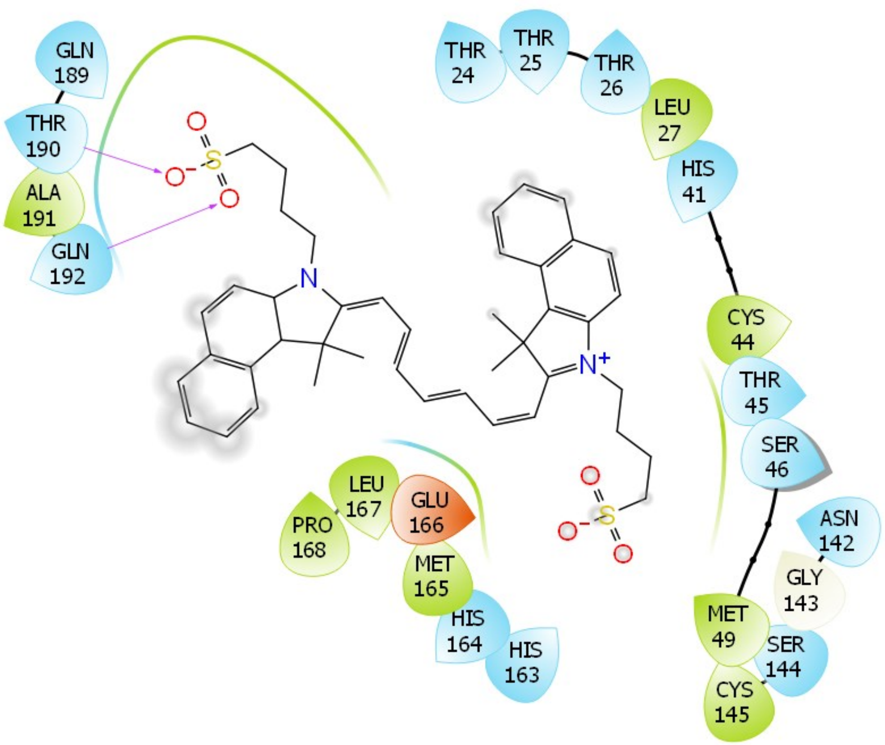	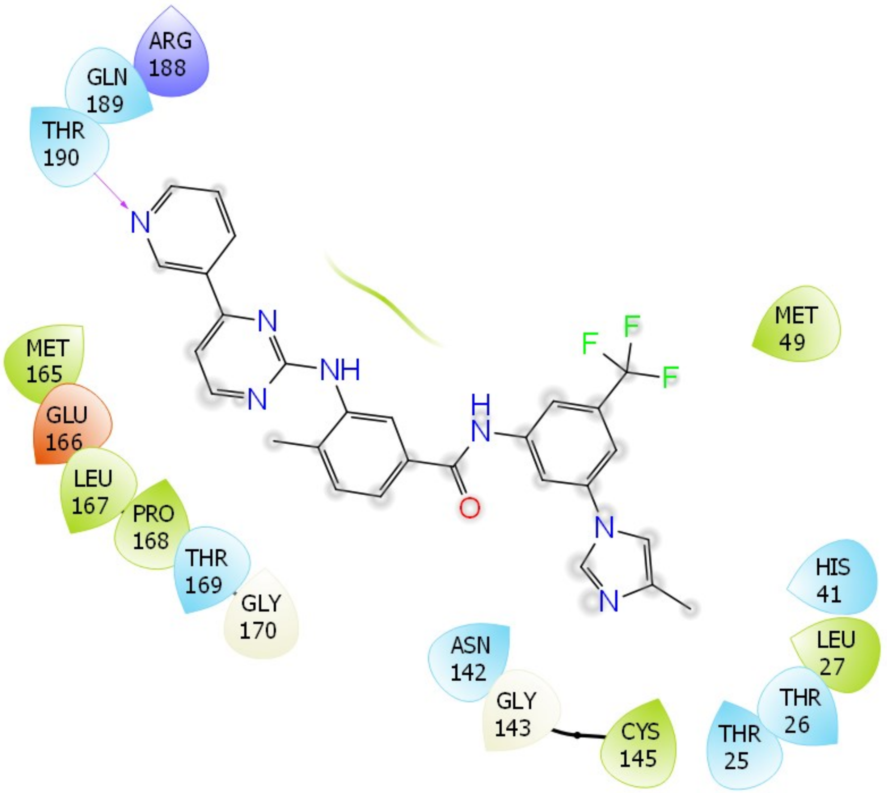

ZINC000064033452

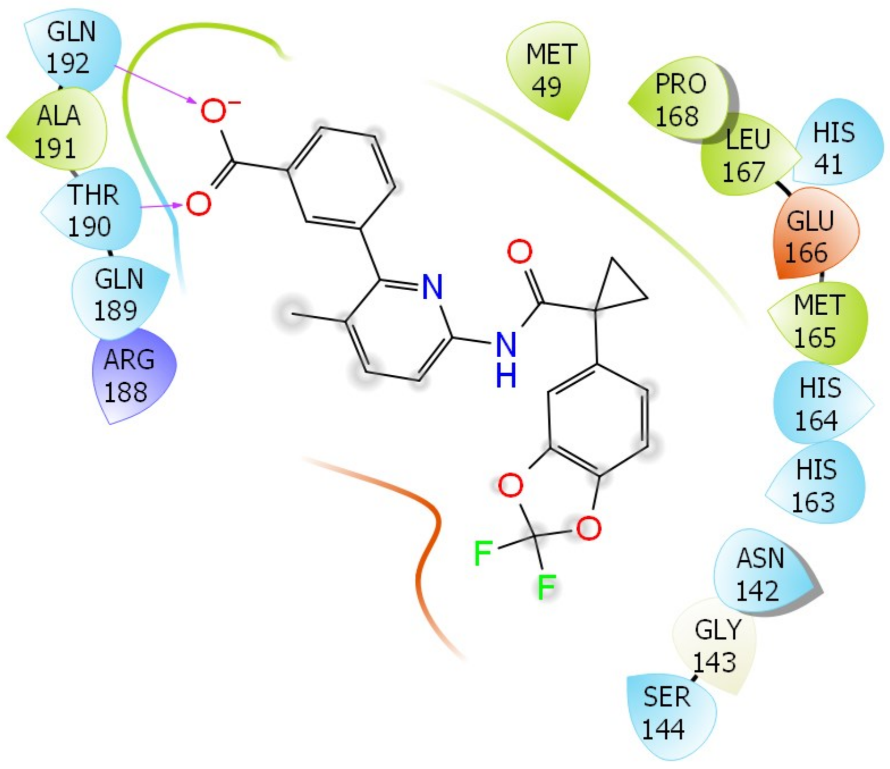

ZINC000164528615

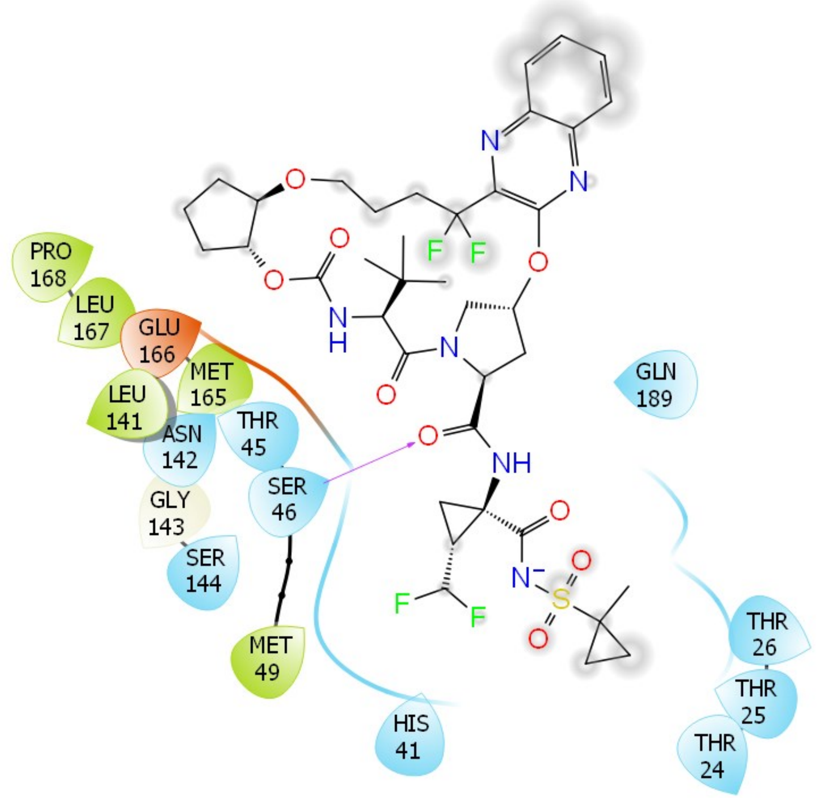

ZINC000052955754	ZINC000004099009
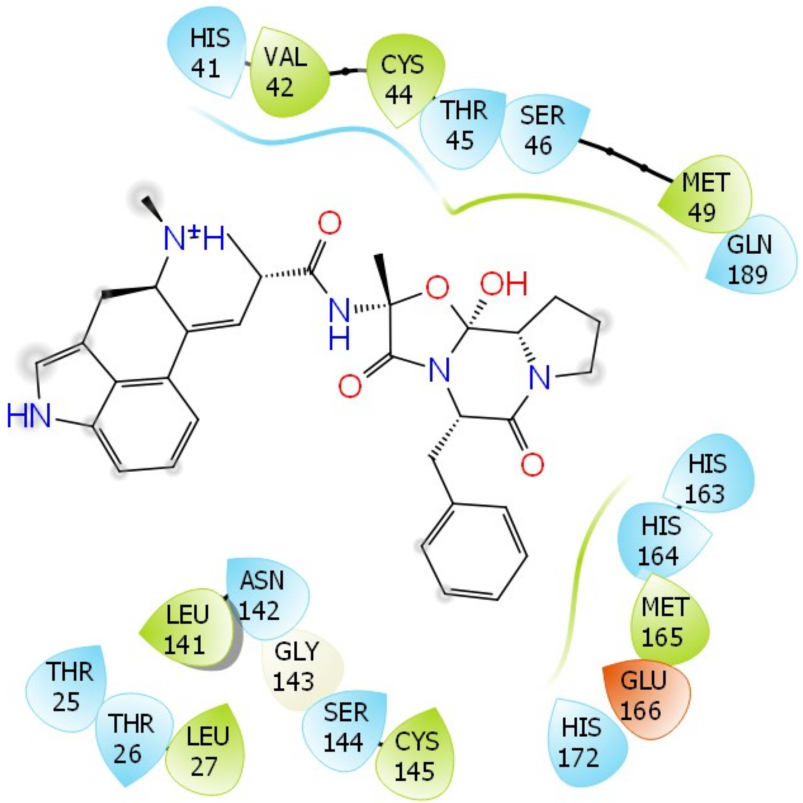	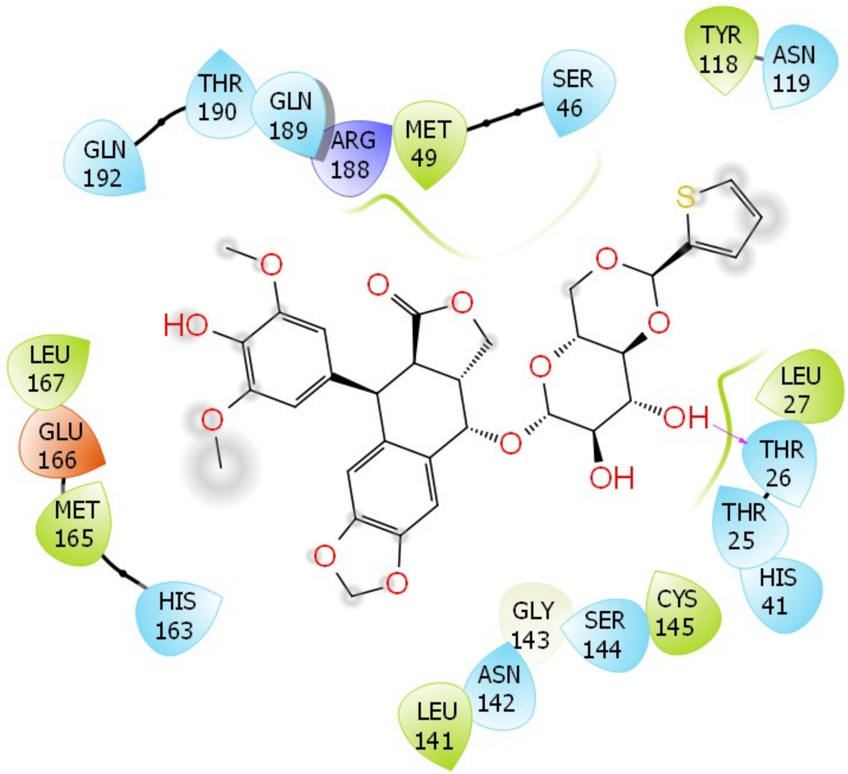

ZINC000006745272	ZINC000084668739
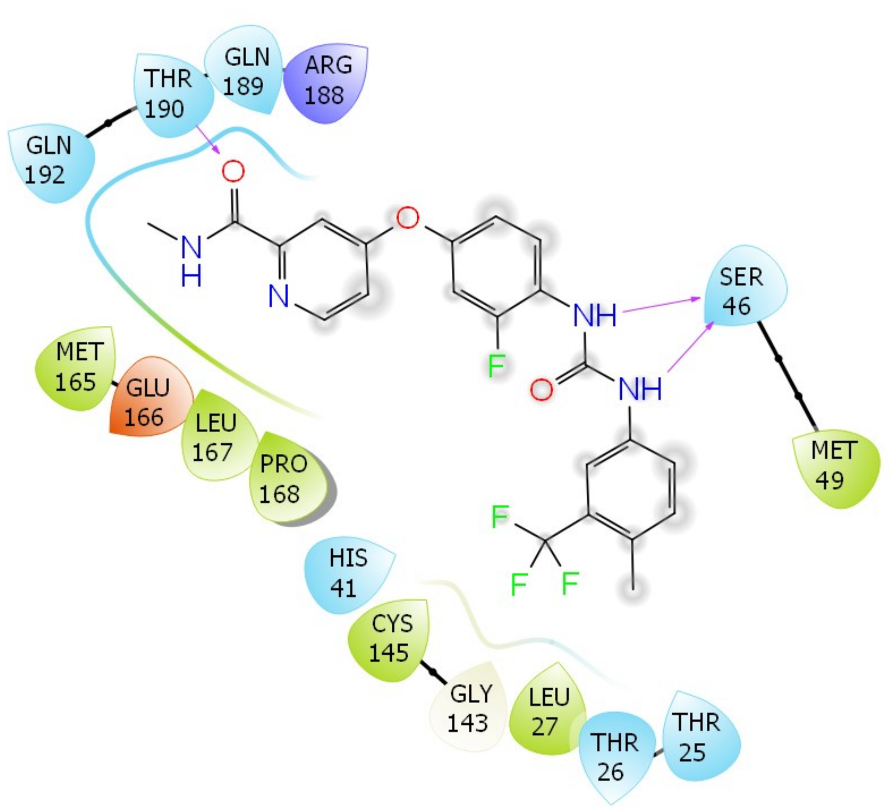	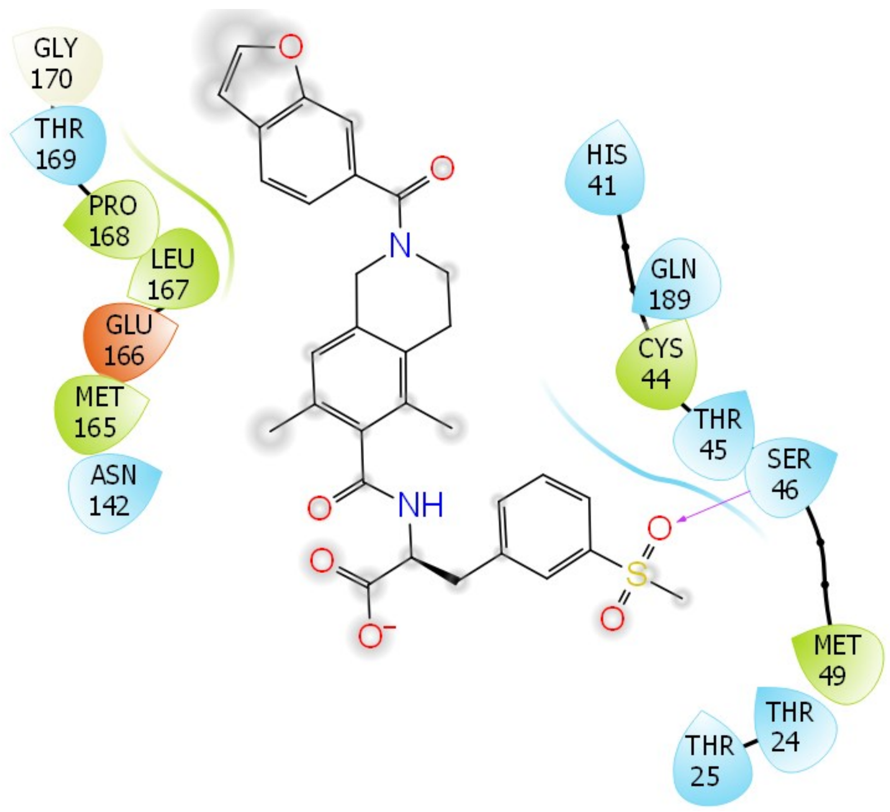

ZINC000019632618	ZINC000169621200
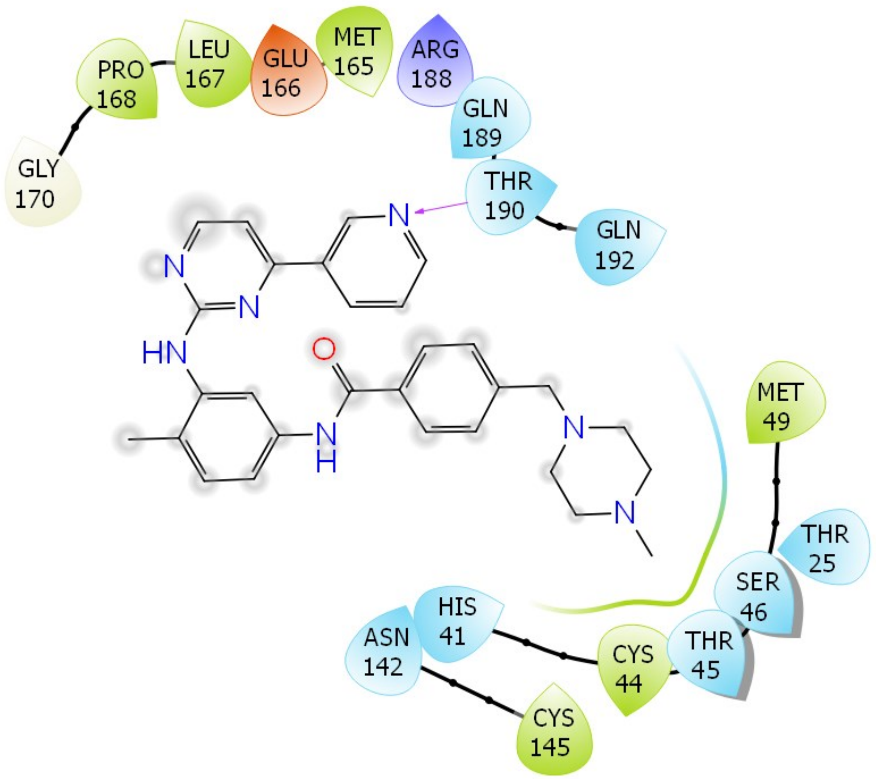	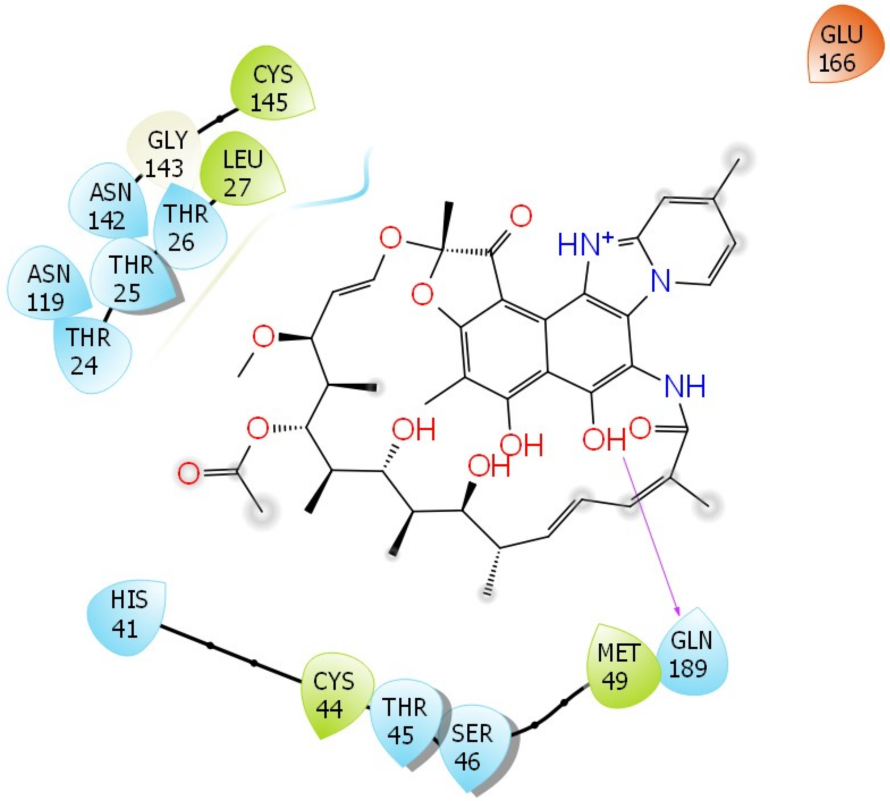

ZINC000100378061	ZINC000014210642
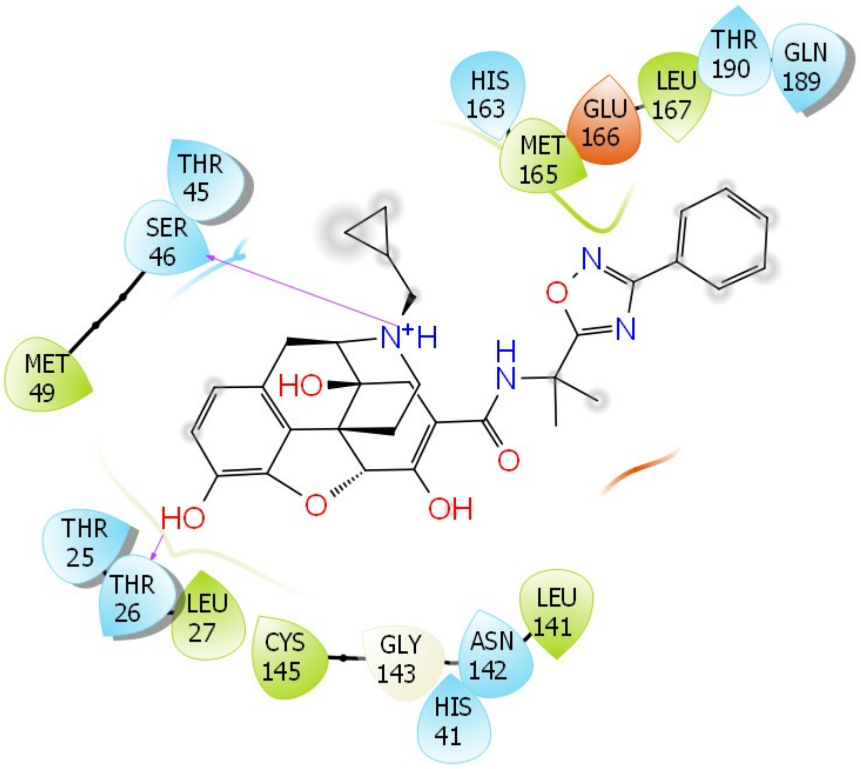	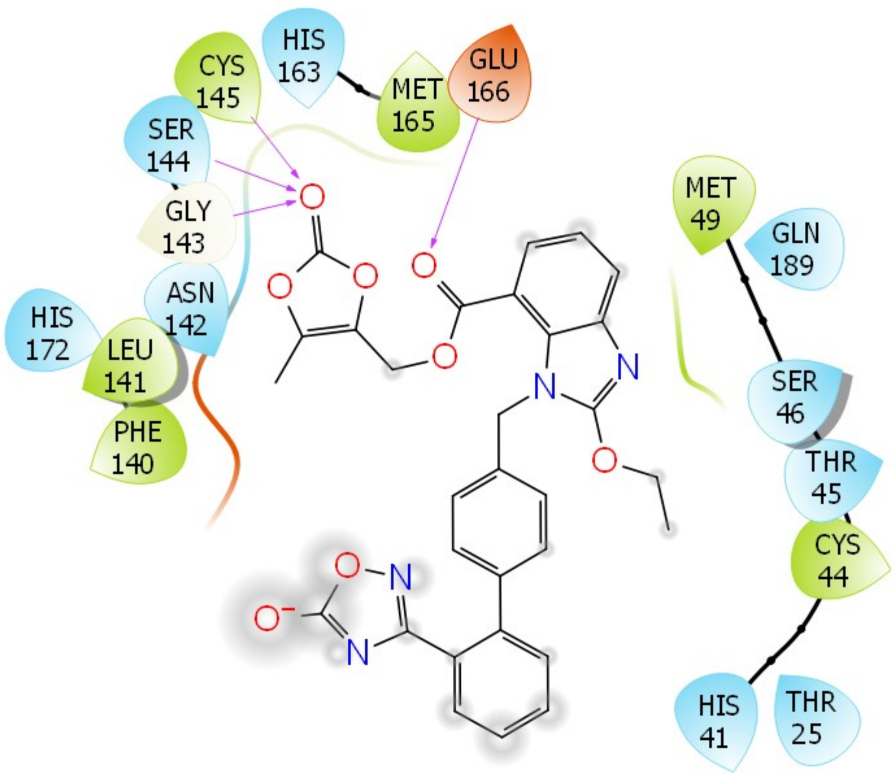

ZINC000058581064

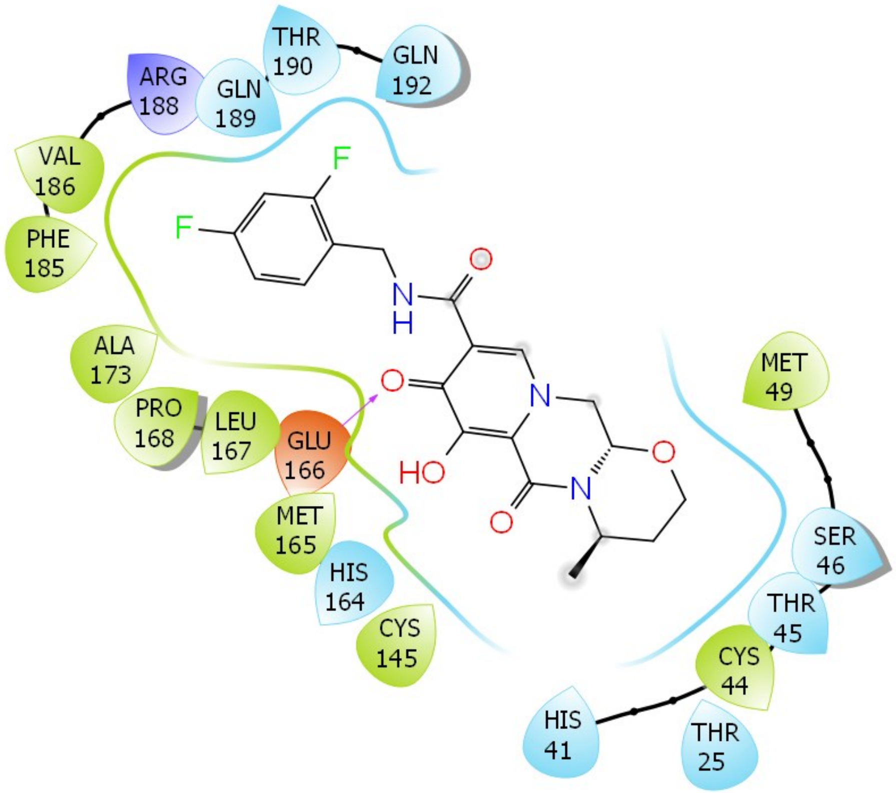

ZINC000003989268

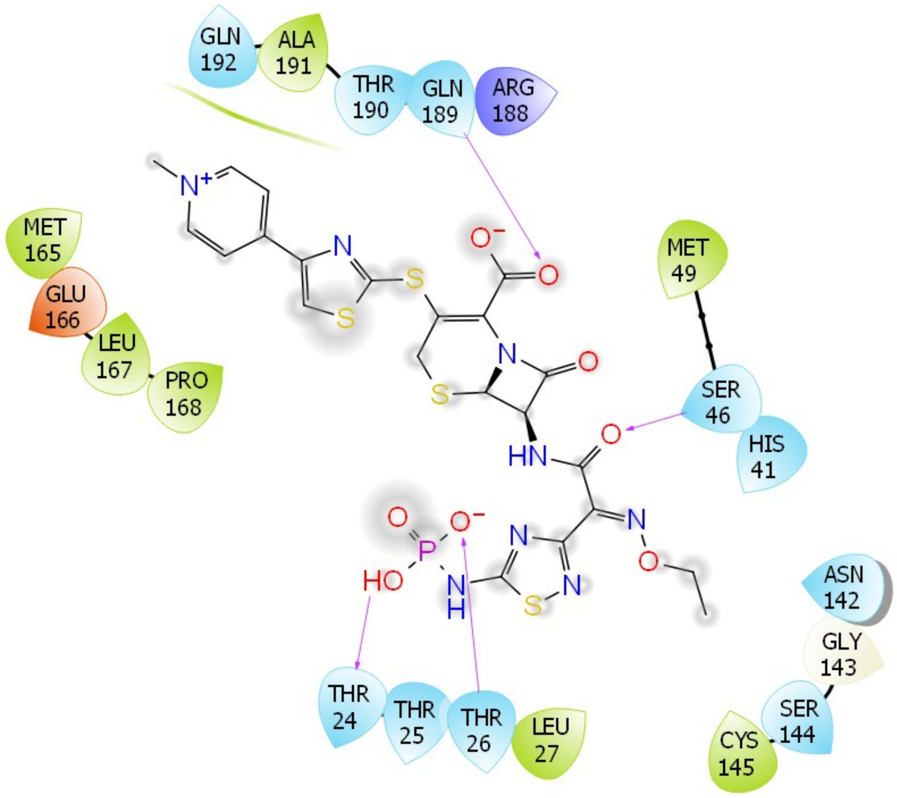

ZINC000029416466	ZINC000068204830
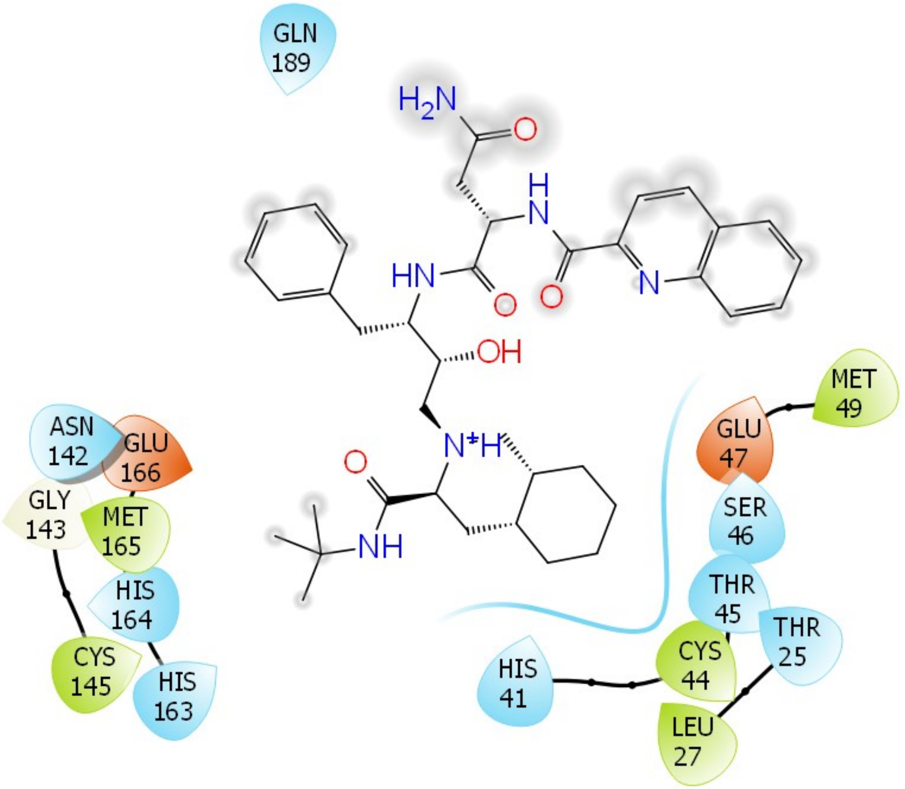	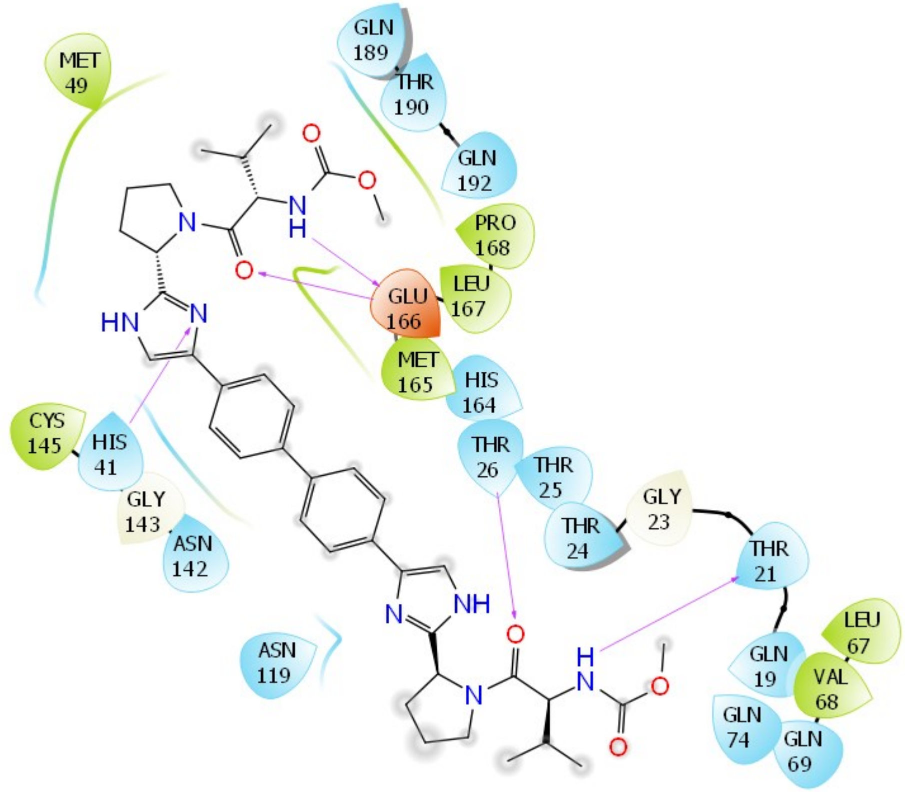

ZINC000070466416

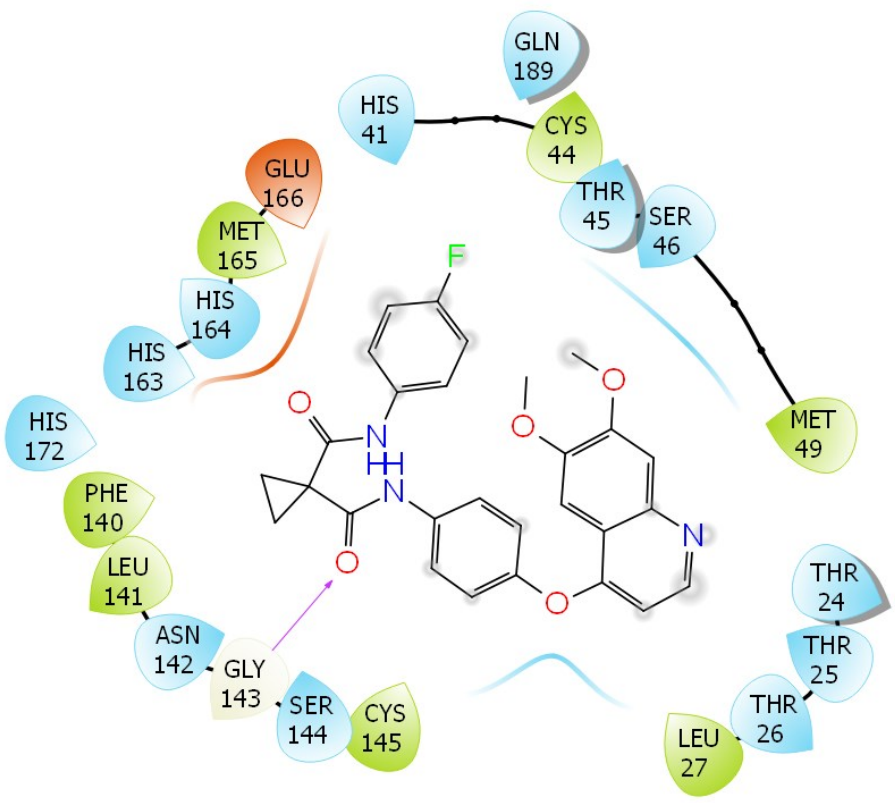

ZINC000013831130

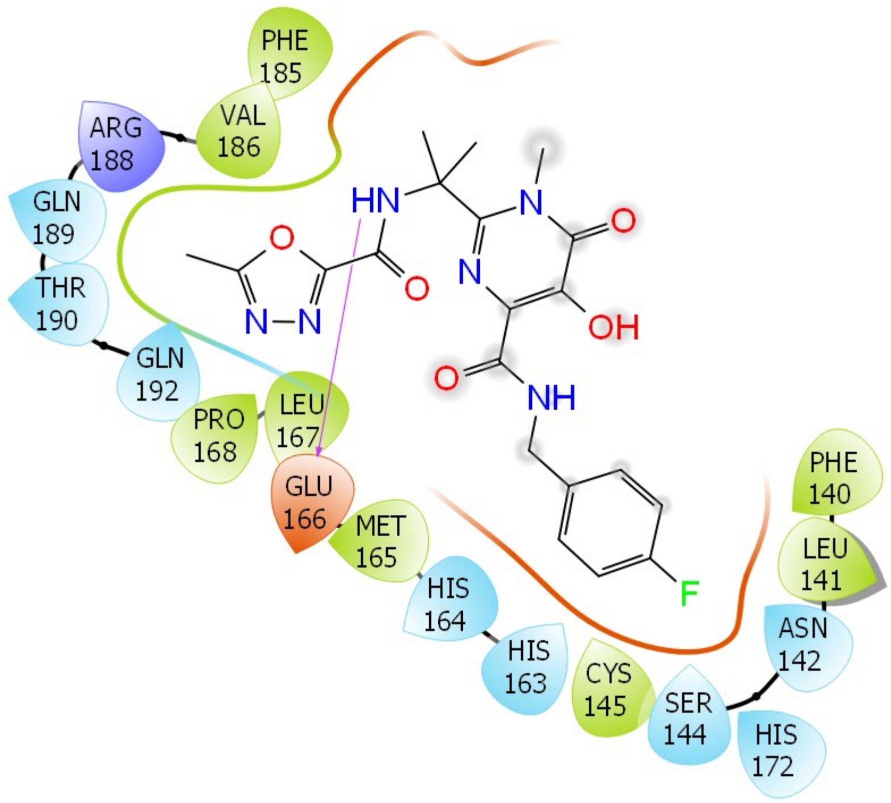

ZINC000094566092	ZINC000009212427
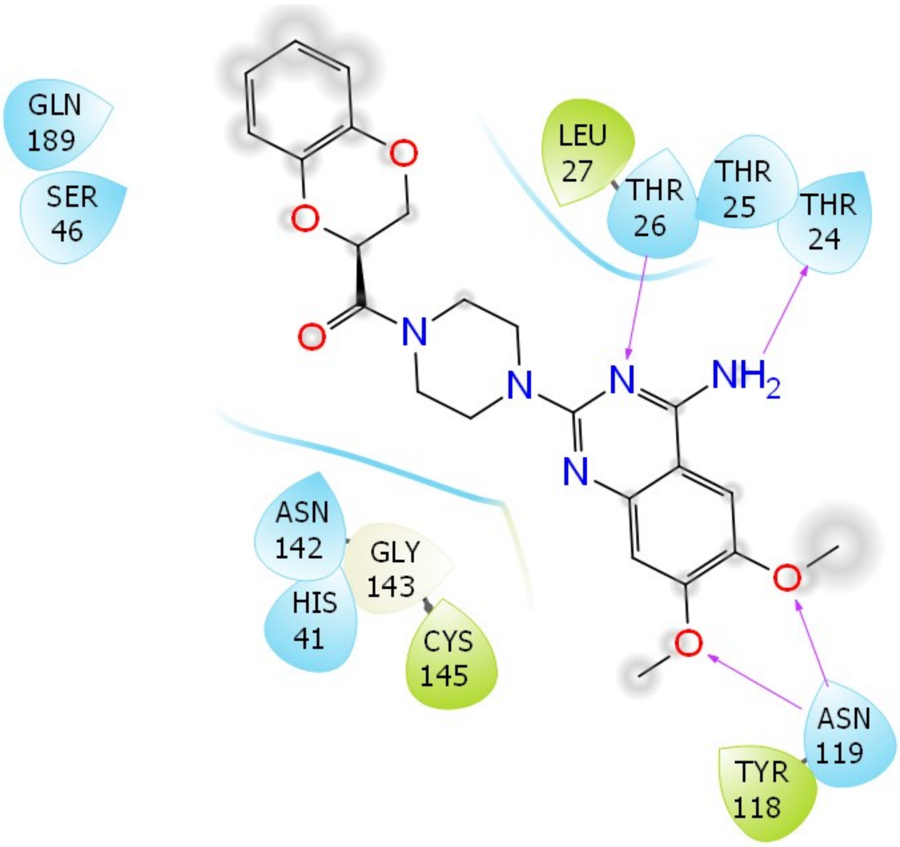	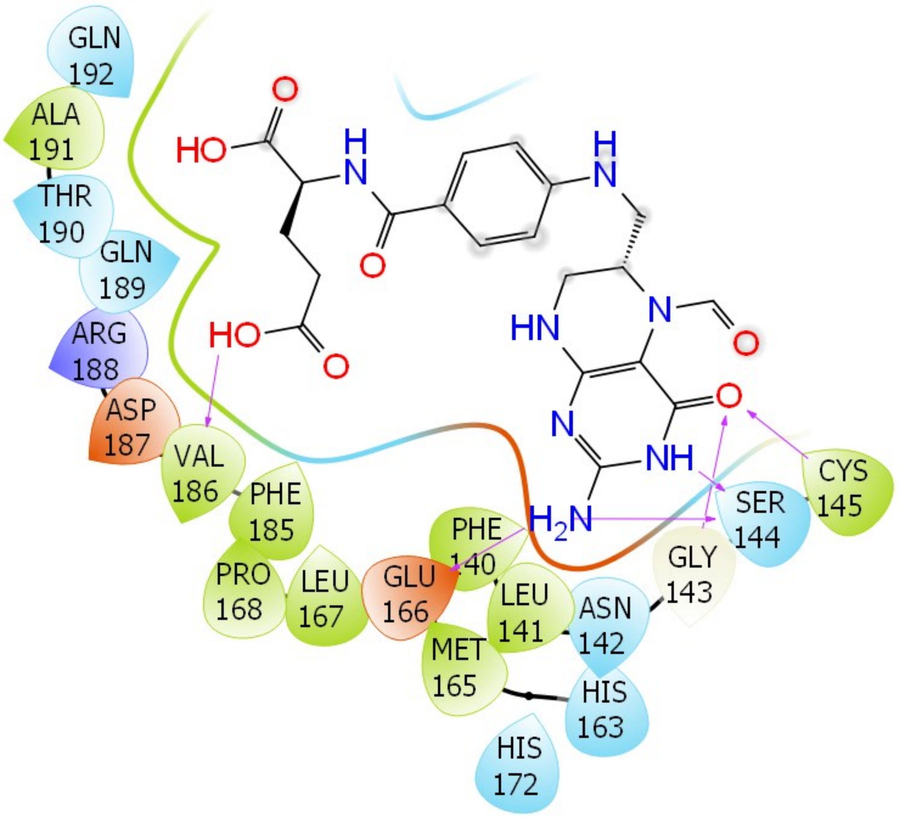

ZINC000003938684	ZINC000003932831
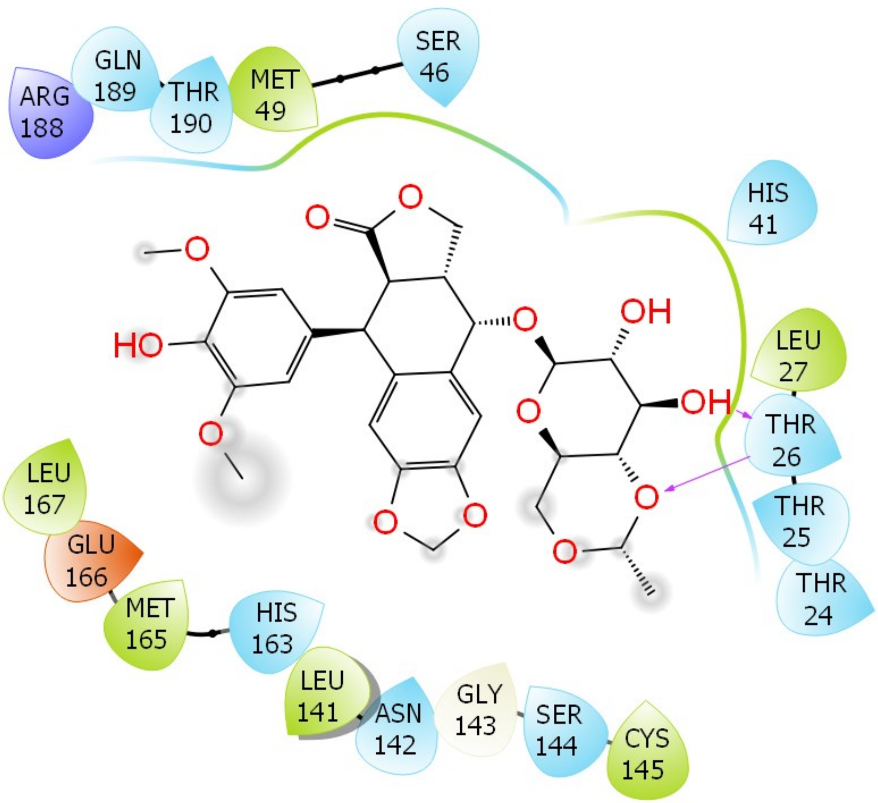	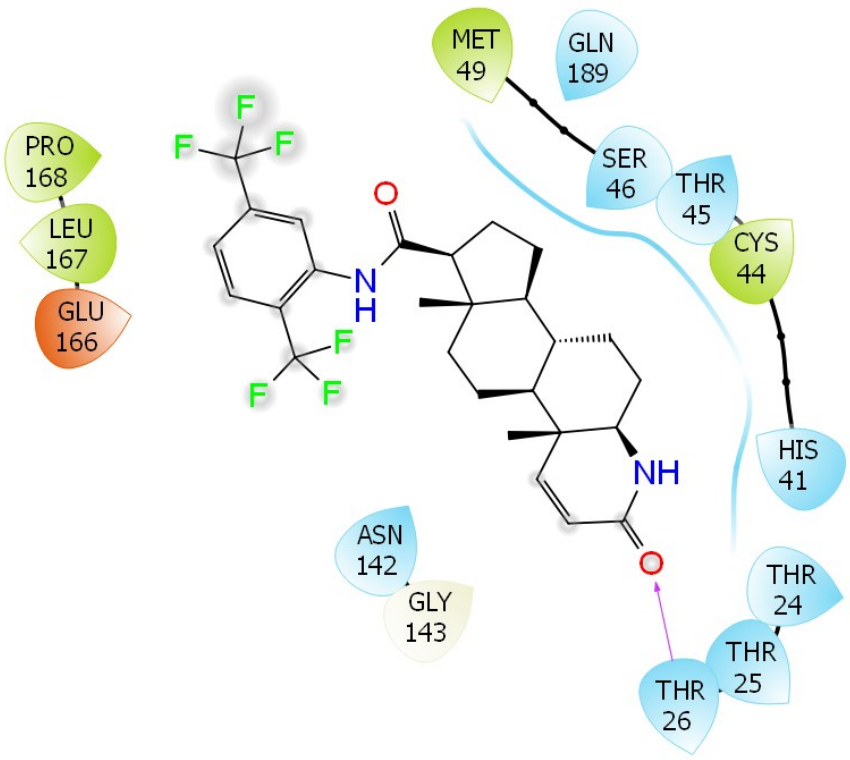

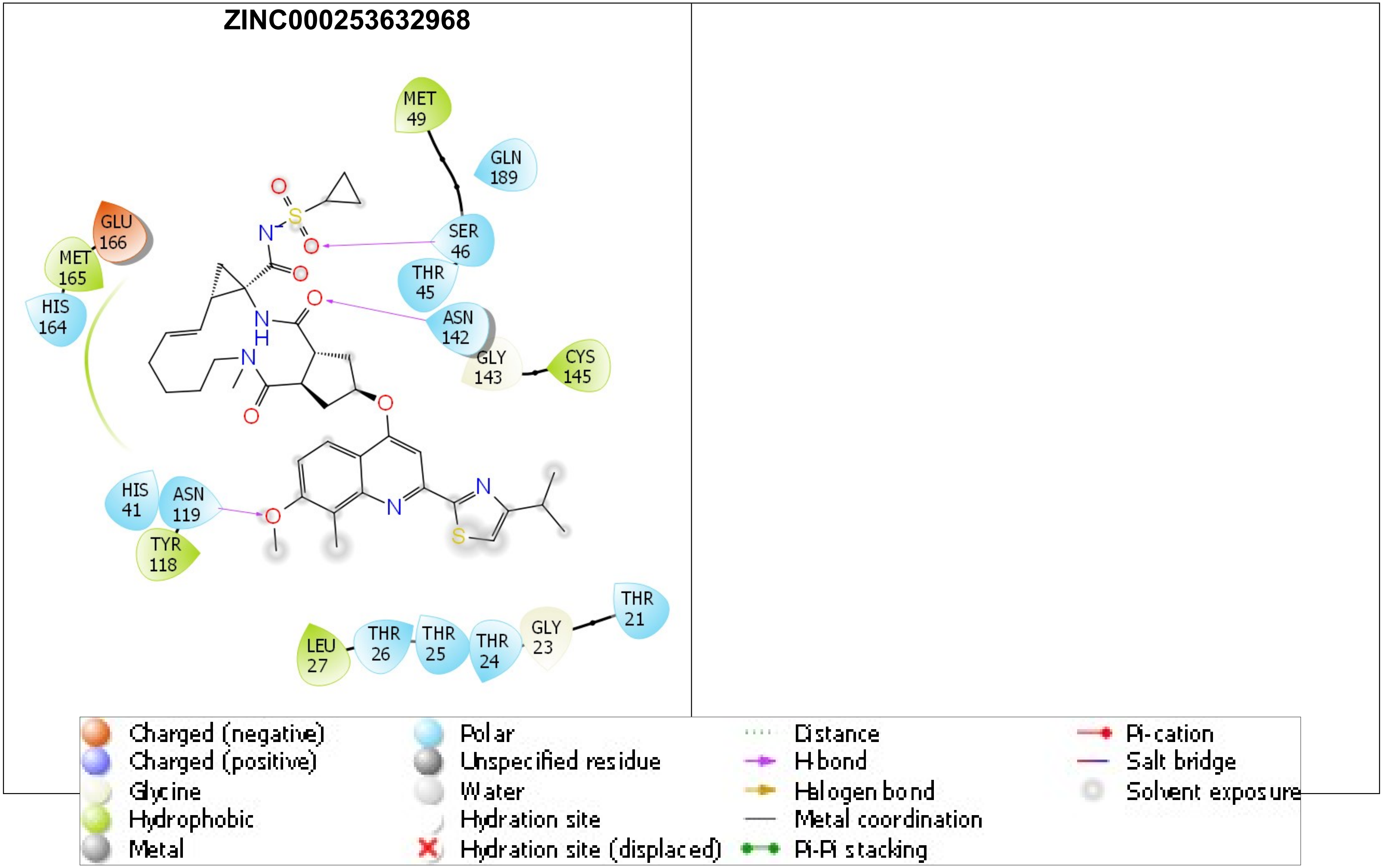

Figure S1. The detailed interaction of SARS-CoV-2 Mpro and the full top-lead compounds

The results are illustrated by the molecular modeling software Maestro, Schrödinger. Hydrogen bonds formed by residues of SARS-CoV-2 Mpro and ligands are indicated by purple arrows. Atoms of carbon, oxygen, nitrogen, and sulfur are presented in black, red, blue, and yellow, respectively.

ZINC000169289767

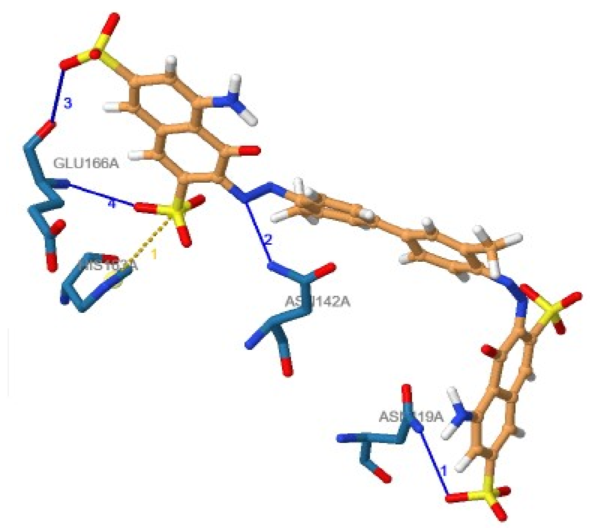

ZINC000001612996

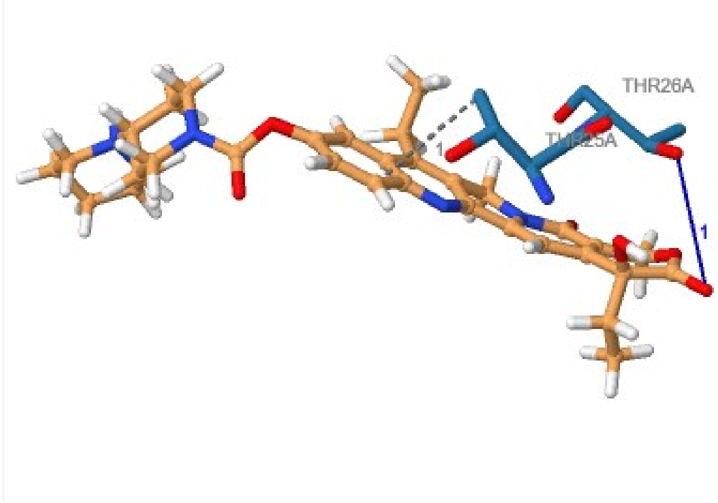

ZINC000000896717

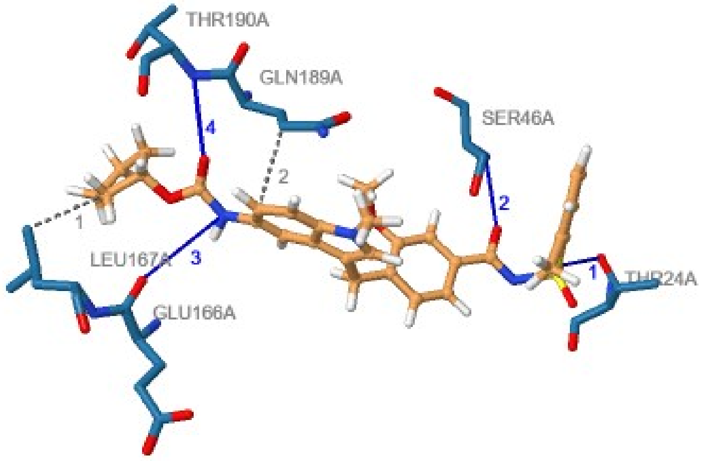

ZINC000003978005

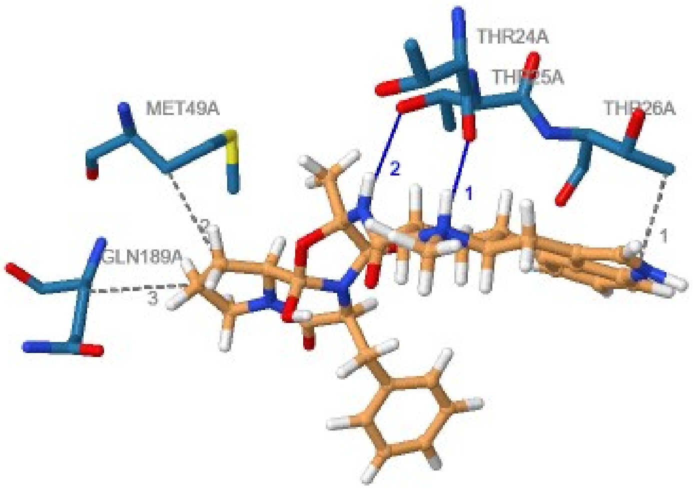

ZINC000008101127	ZINC000006716957
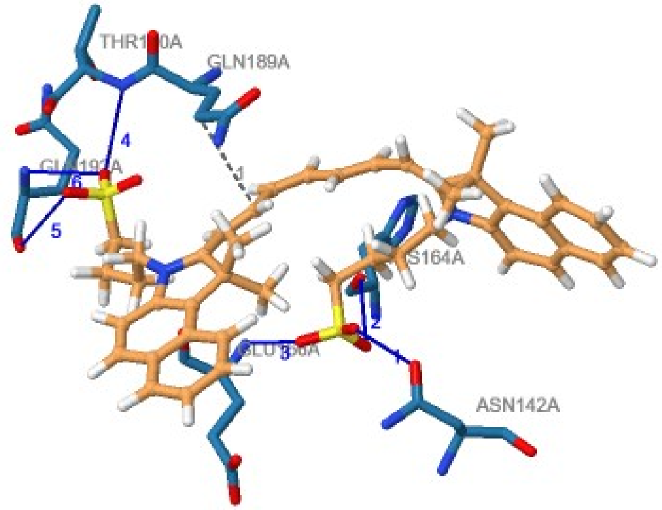	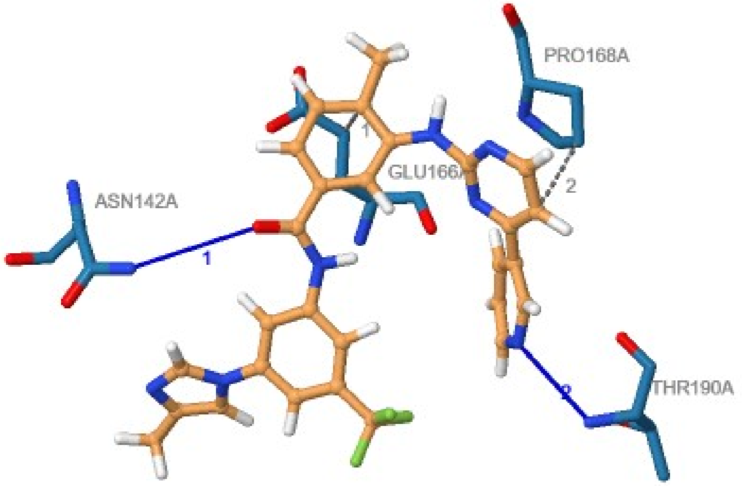

ZINC000064033452	ZINC000164528615
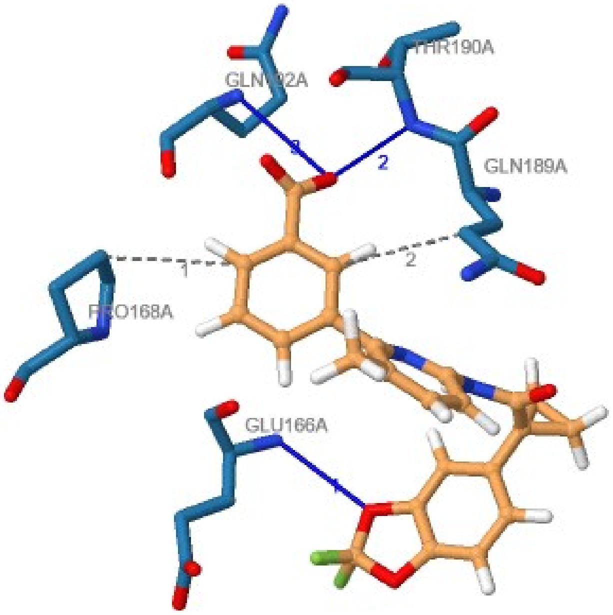	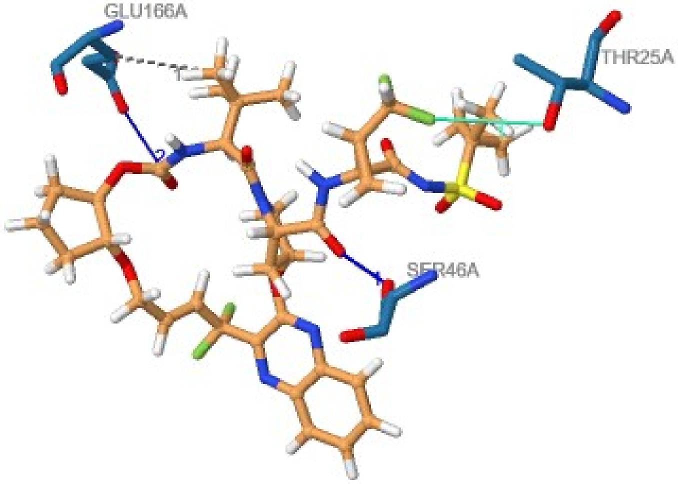

ZINC000052955754

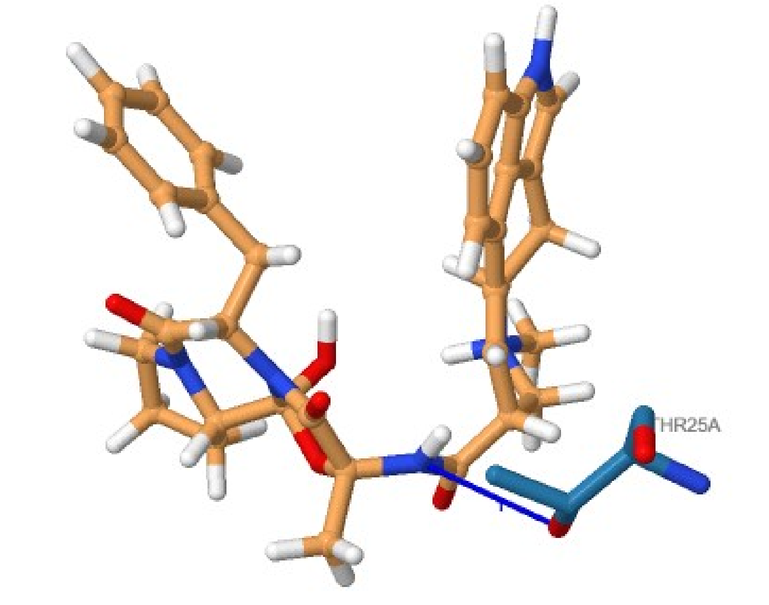

ZINC000004099009

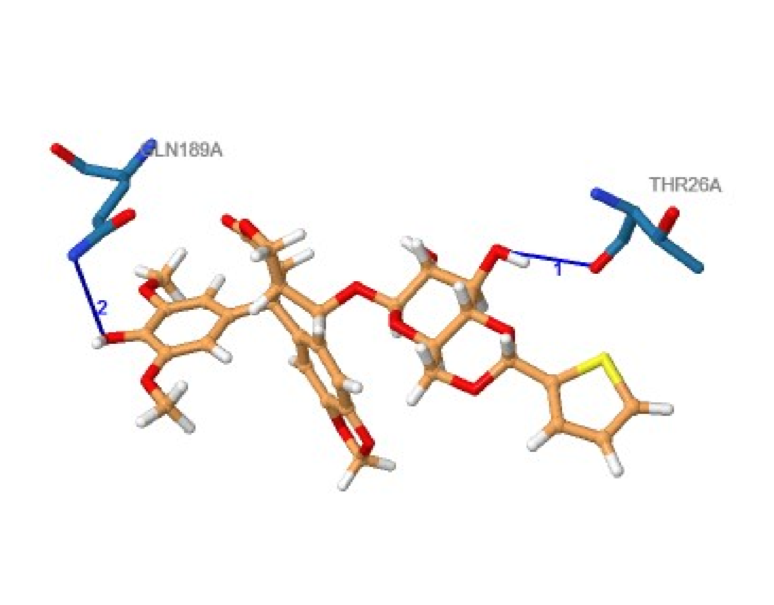

ZINC000006745272

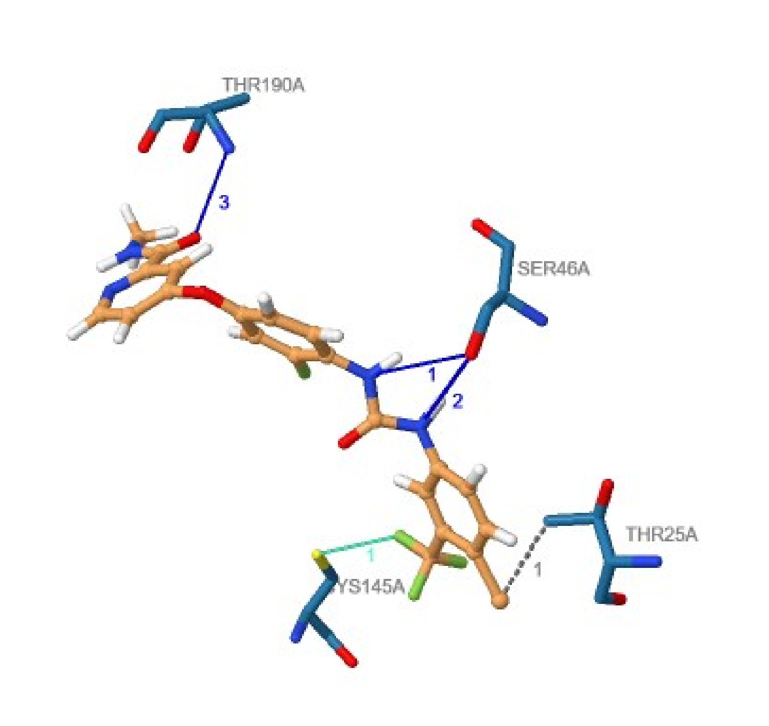

ZINC000084668739

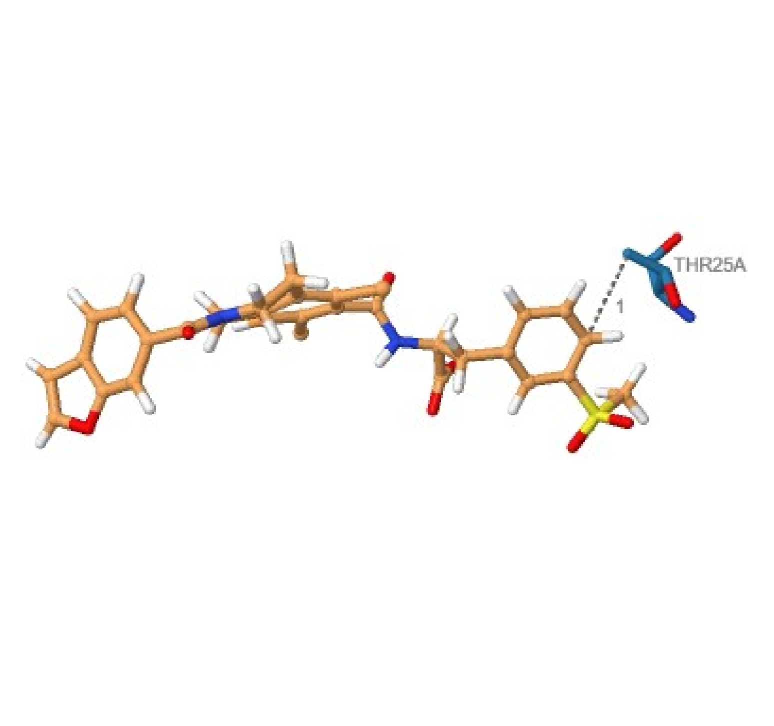

ZINC000019632618	ZINC000169621200
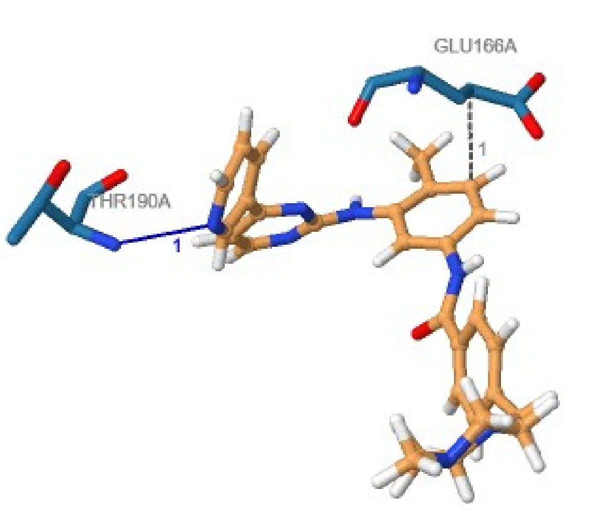	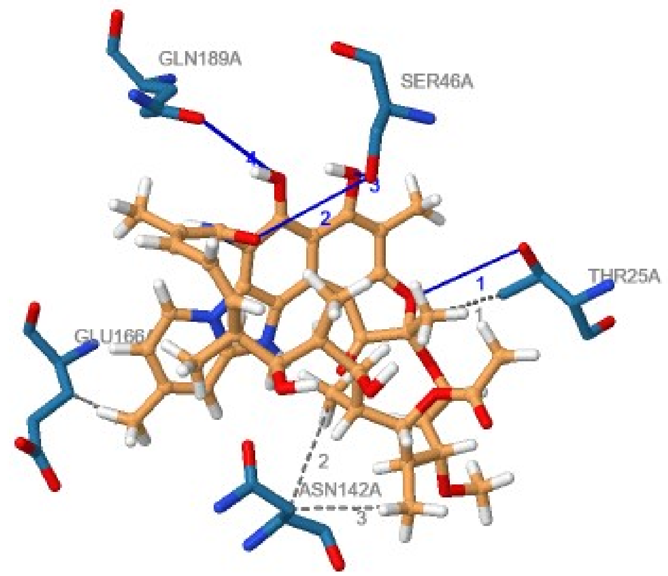

ZINC000100378061	ZINC000014210642
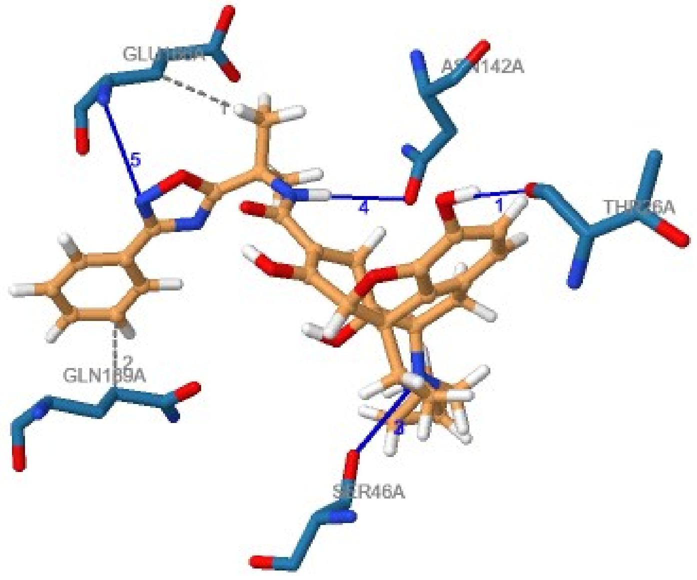	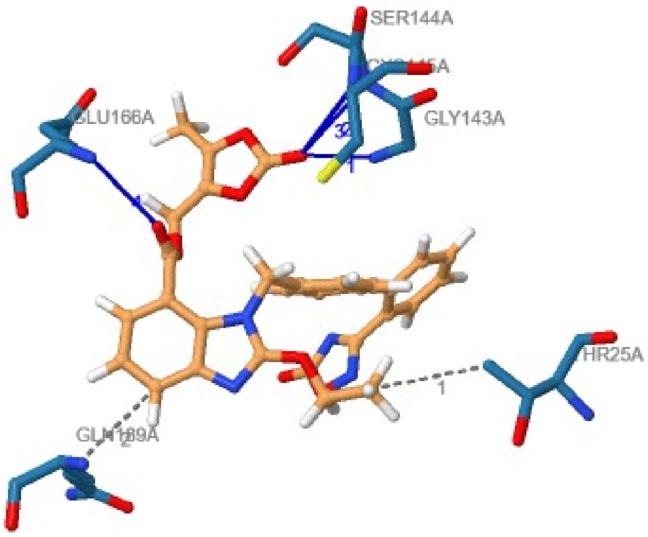

ZINC000058581064

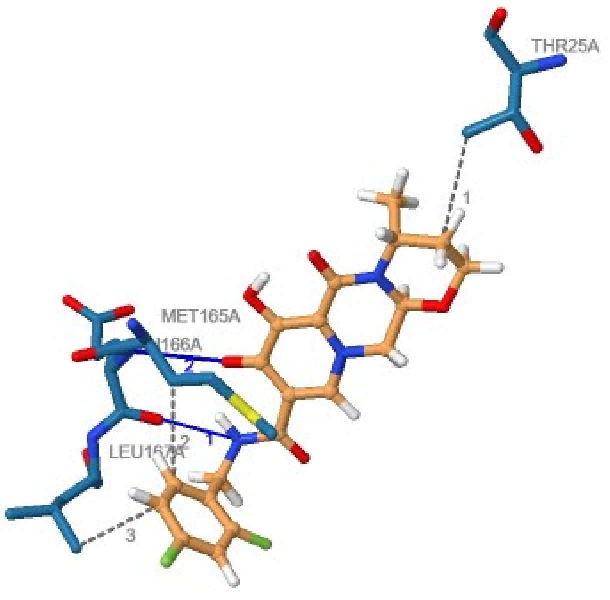

ZINC000003989268

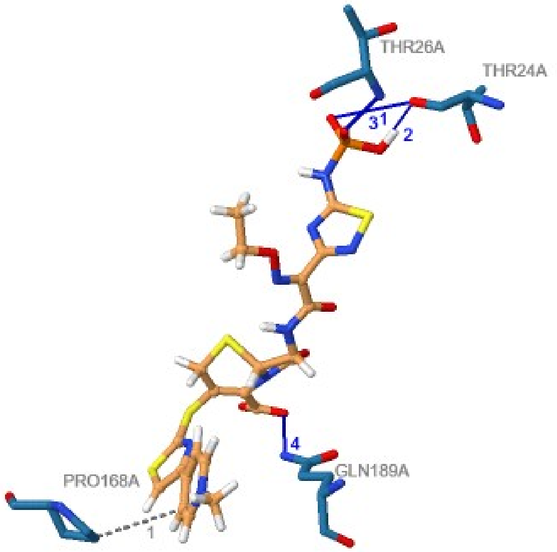

ZINC000029416466

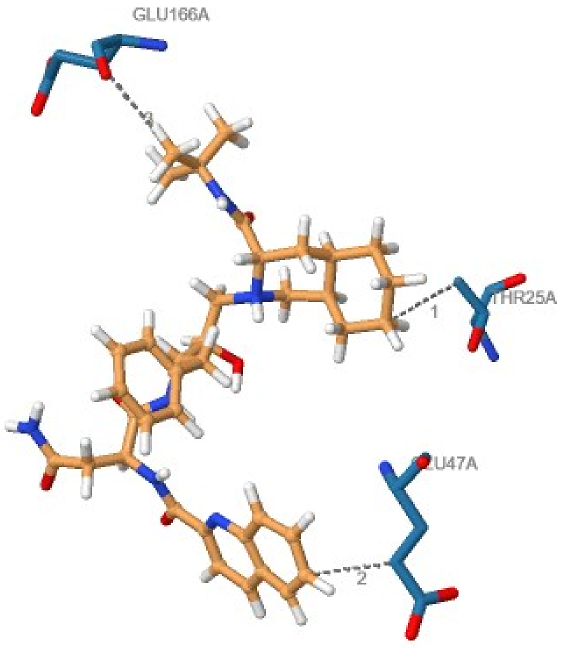

ZINC000068204830

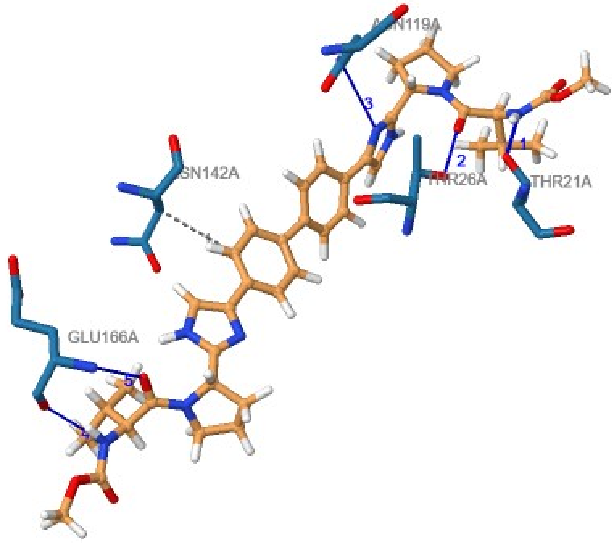

ZINC000070466416

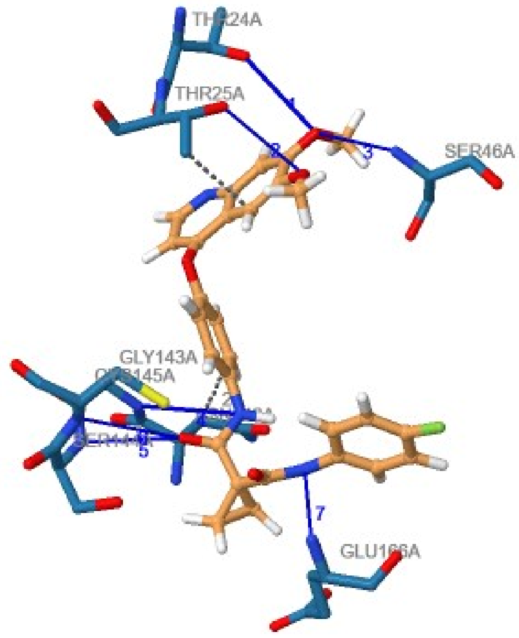

ZINC000013831130

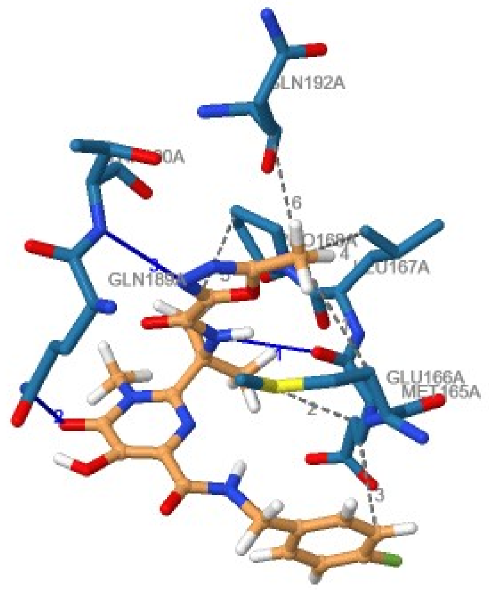

ZINC000094566092

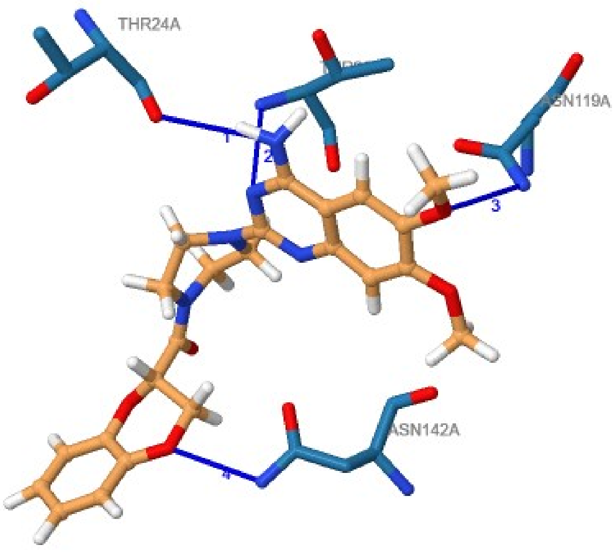

ZINC000009212427

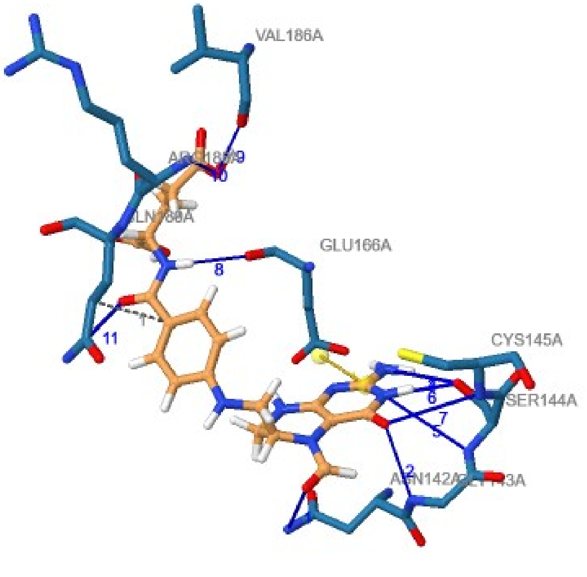

ZINC000003938684

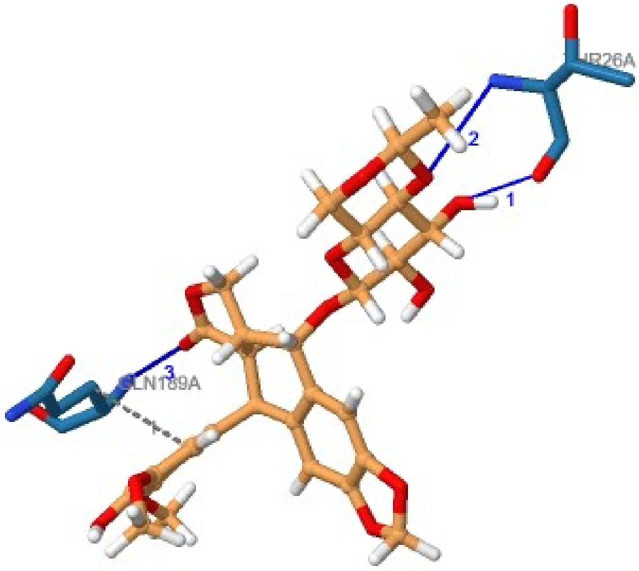

ZINC000003932831

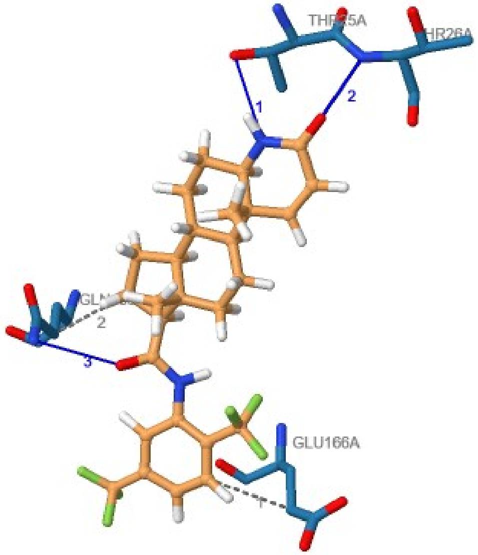

ZINC000253632968

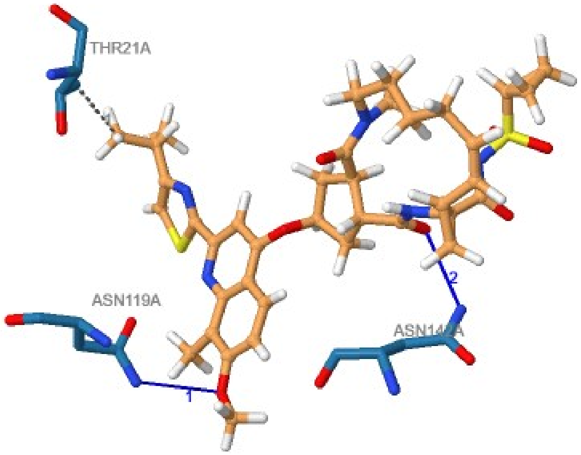

Figure S2. The dominant conformation of the top-lead compounds in the complex with SARS-CoV-2 Mpro

The result was obtained by all-atom clustering with a cut-off of 0.3 nm over the last NPT. Blue and green lines indicate hydrogen and halogen bonds between the ligand and the residues of SARS-CoV-2 Mpro, respectively. Grey and yellow dots indicate hydrophobic interactions and salt bridges between the ligand and the residues of SARS-CoV-2 Mpro, respectively. The figure was generated using a PLIP web service.[1]

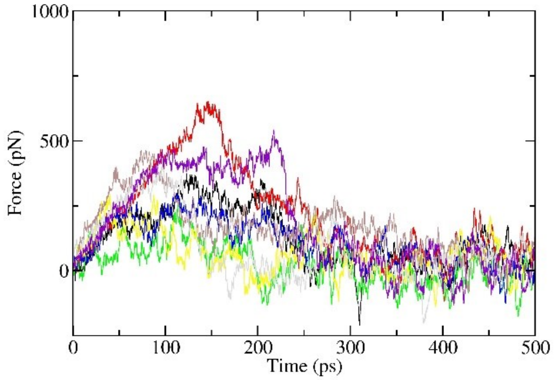

ZINC000169289767

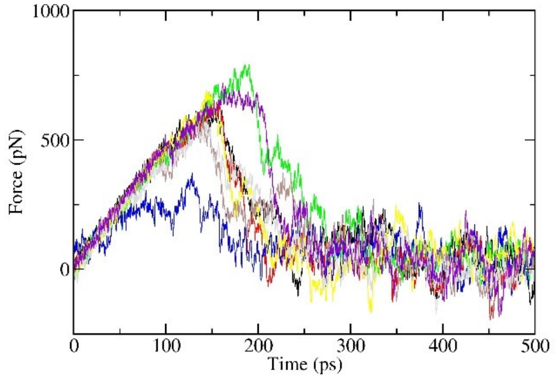

ZINC000001612996

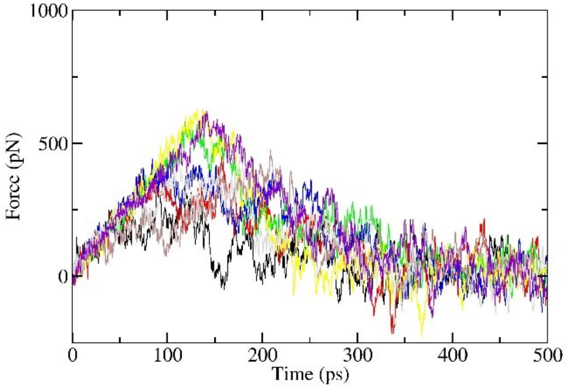

ZINC000000896717

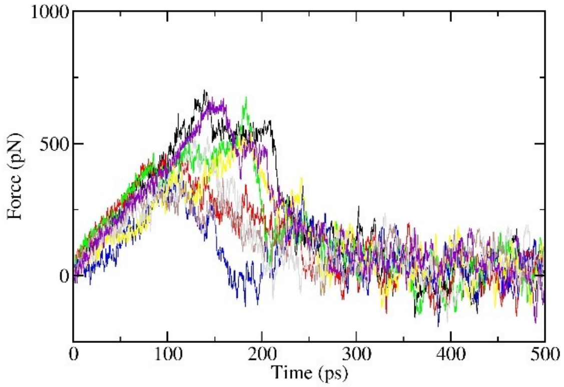

ZINC000003978005

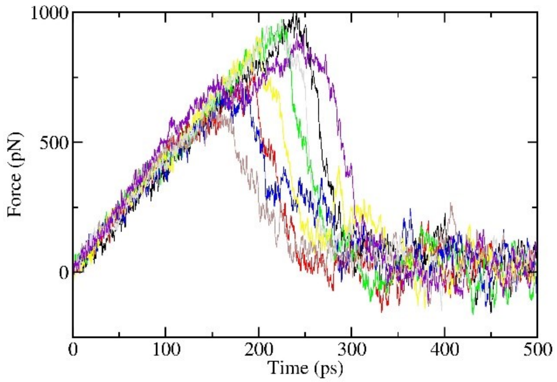

ZINC000008101127

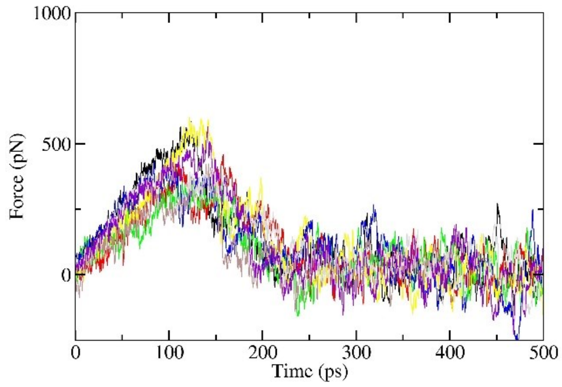

ZINC000006716957

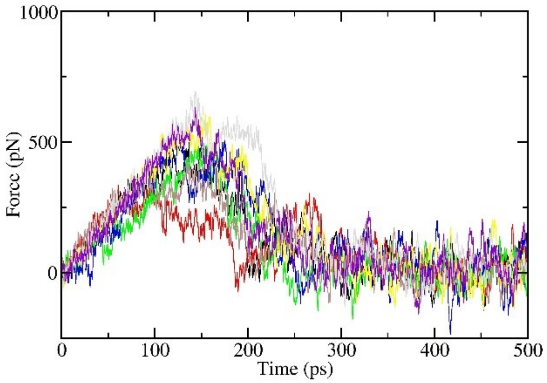

ZINC000064033452

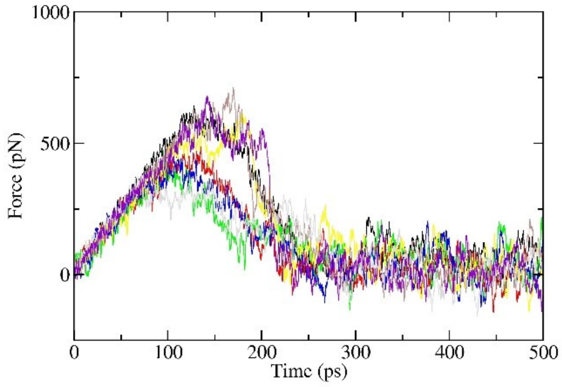

ZINC000164528615

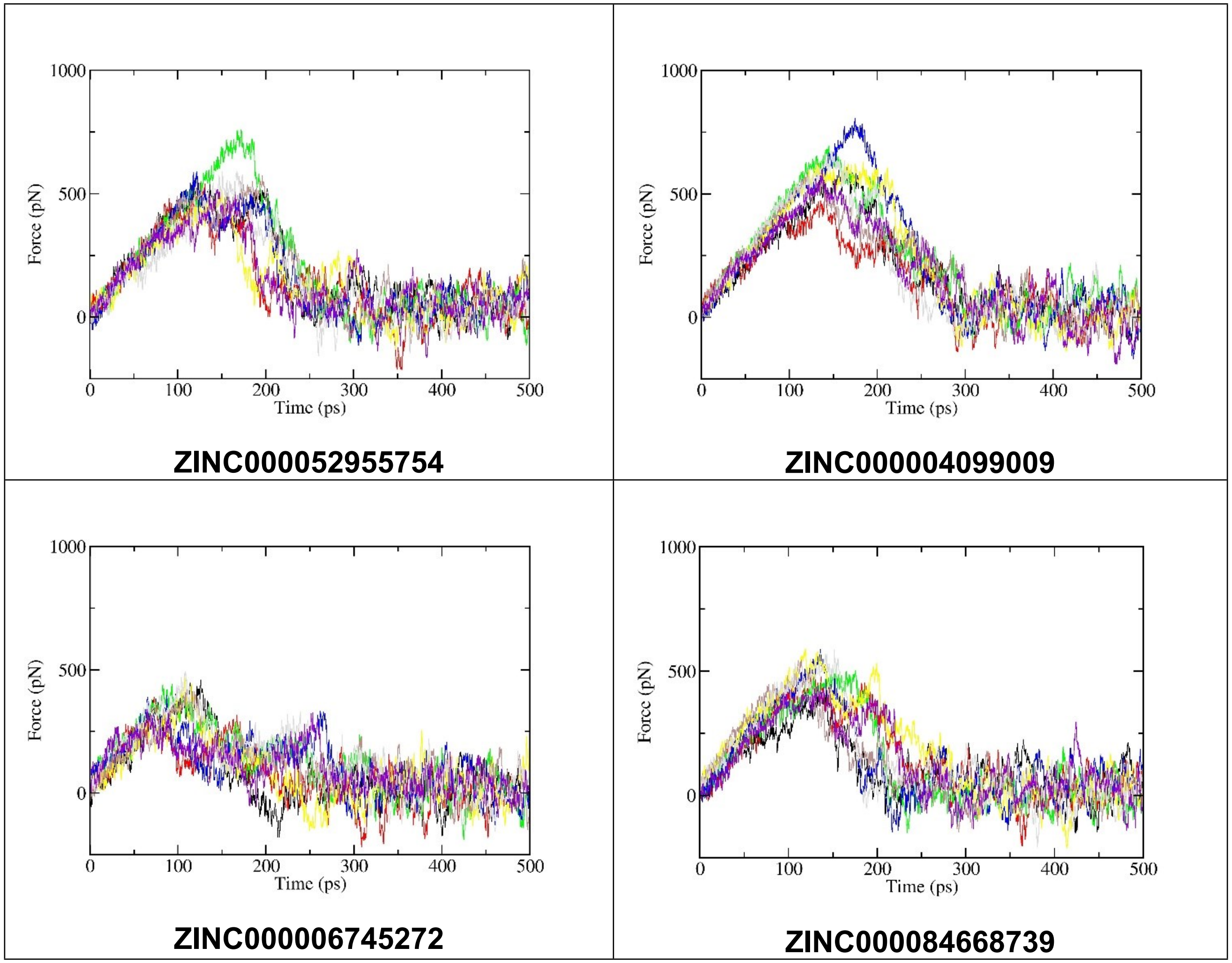

Force (pN)
Time (ps)
ZINC000052955754
ZINC000004099009
ZINC000006745272
ZINC000084668739

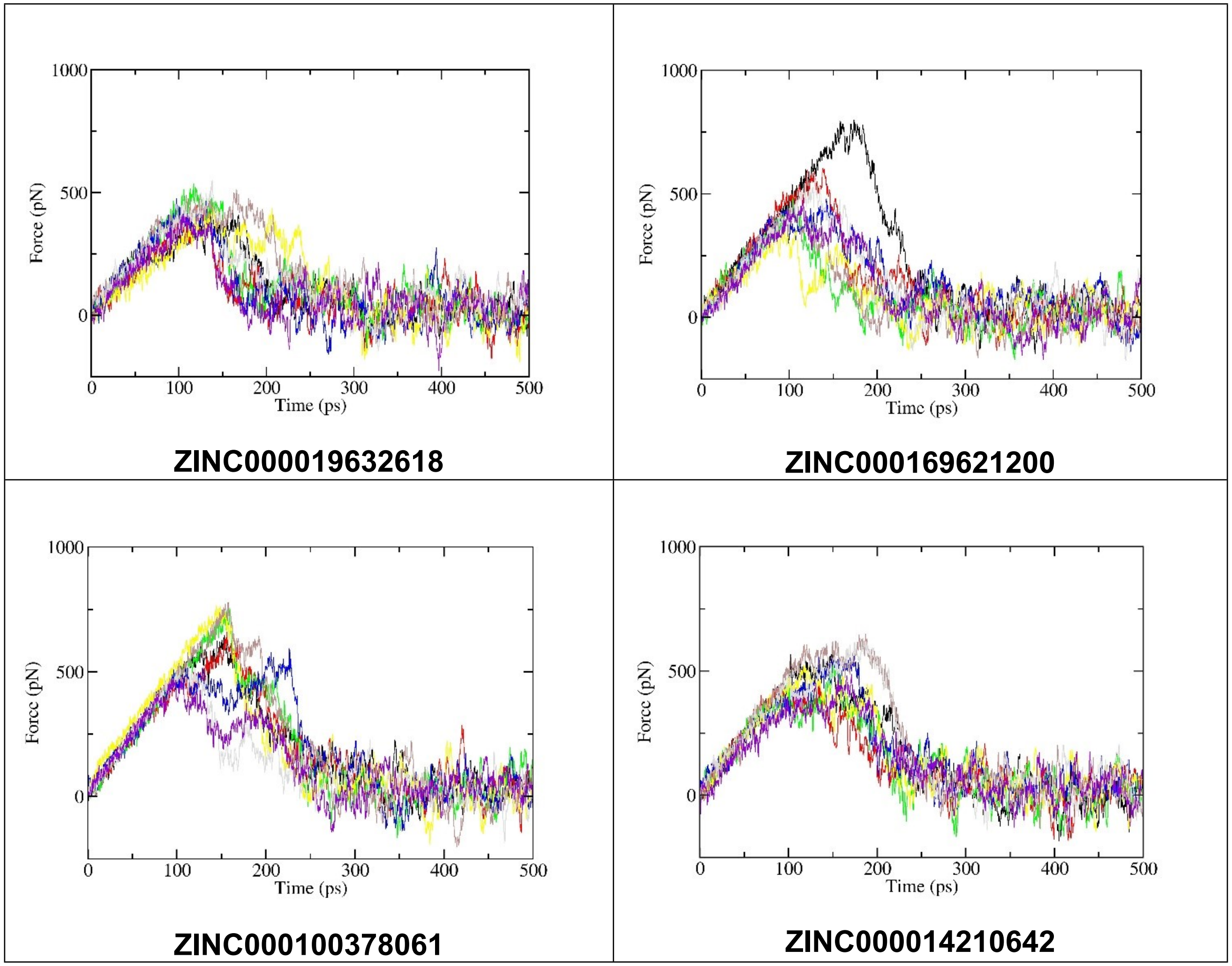
Force (pN)
Time (ps)
0
100
200
300
400
500
1000
ZINC000019632618
ZINC000169621200
ZINC000100378061
ZINC000014210642

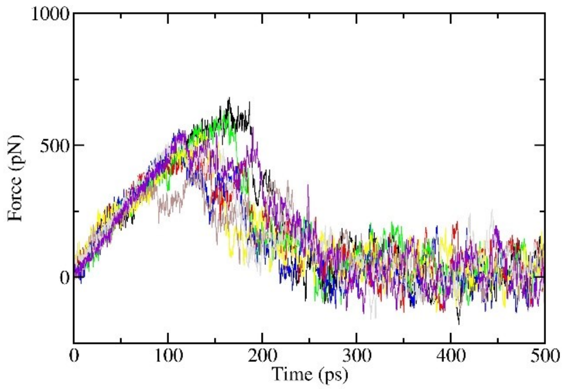

ZINC000058581064

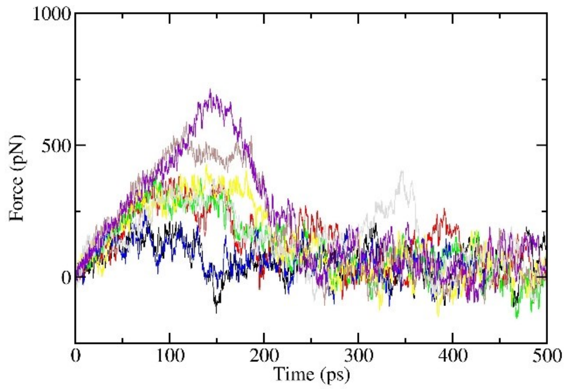

ZINC000003989268

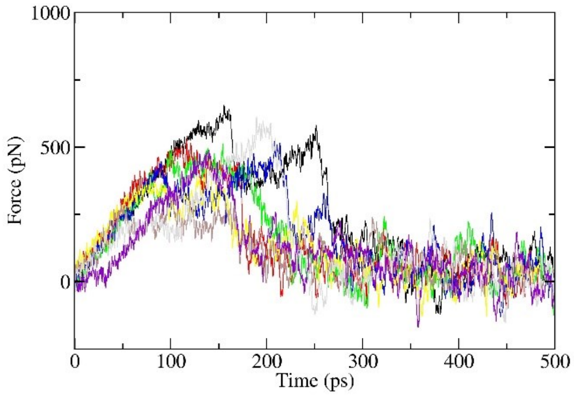

ZINC000029416466

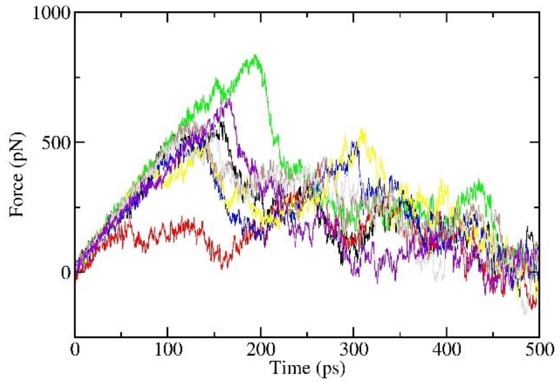

ZINC000068204830

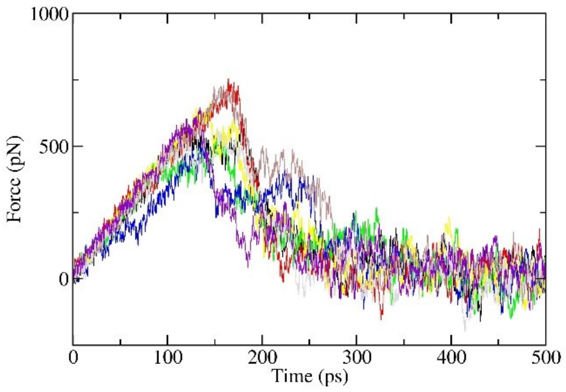

ZINC000070466416

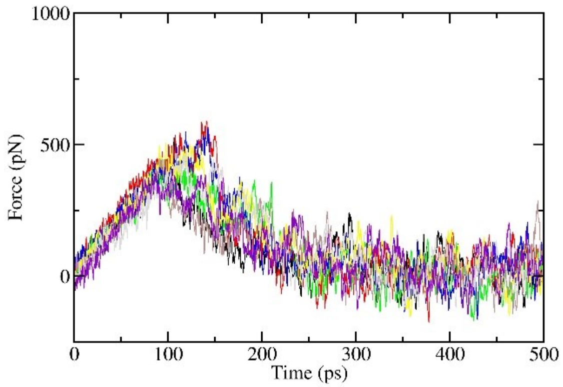

ZINC000013831130

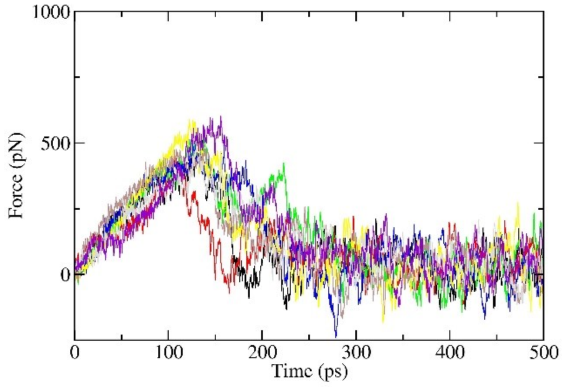

ZINC000094566092

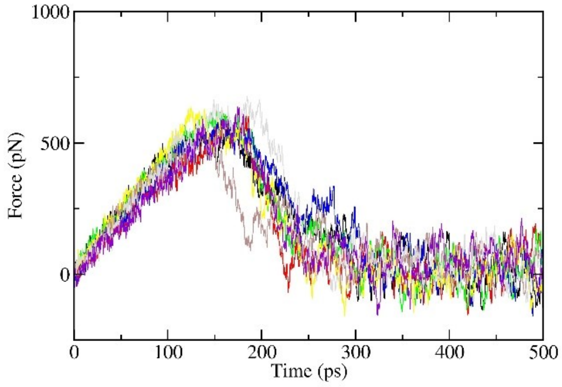

ZINC000009212427

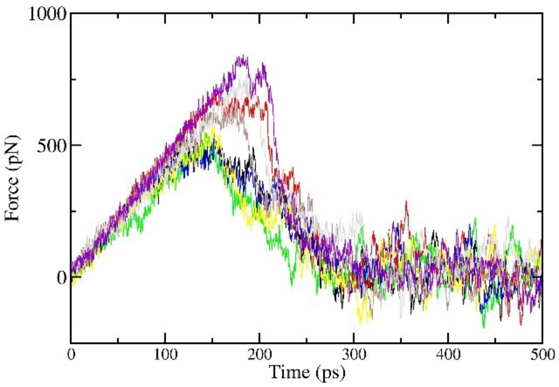

ZINC000003938684

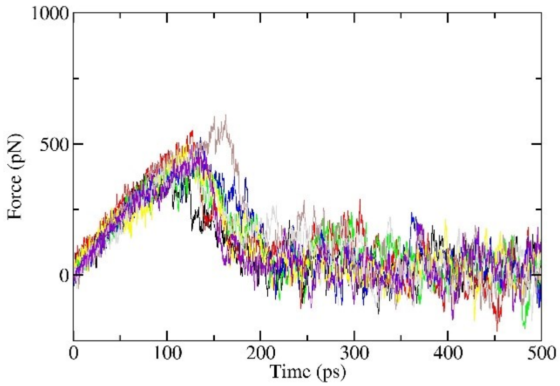

ZINC000003932831

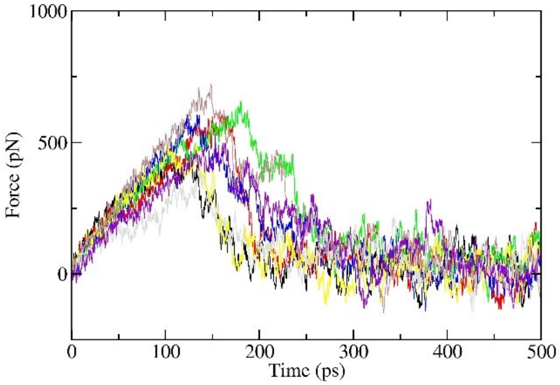

ZINC000253632968

Figure S3. The pulling force in time-dependence over 8 independent SMD trajectories. Each interaction force is reported every 0.02 ps and is the 8 independent trajectories average value. Each interaction work is reported every 10 ps and is the 8 independent trajectories average value. The figure was prepared using PyMOL 1.3 open source.

References

1. S. Salentin, S. Schreiber, V. J. Haupt, M. F. Adasme and M. Schroeder, *Nucleic acids research*, 2015, **43**, W443-W447.
